# An explainable federated blockchain framework with privacy-preserving AI optimization for securing healthcare data

**DOI:** 10.1038/s41598-025-04083-4

**Published:** 2025-07-01

**Authors:** Tanisha Bhardwaj, K. Sumangali

**Affiliations:** School of Computer Science Engineering and Information Systems, Vellore Institute of Technology, 632014 Vellore, Tamilnadu India

**Keywords:** Blockchain, Consensus Mechanism, Data Protection, Decentralized Learning, Differential Privacy (DP), Federated Learning (FL), Neural Network, Privacy Security, Health care, Engineering

## Abstract

With the rapid growth of healthcare data and the need for secure, interpretable, and decentralized machine learning systems, Federated Learning (FL) has emerged as a promising solution. However, FL models often face challenges regarding privacy preservation, transparency, and resistance to adversarial attacks. To address these limitations, this paper proposes the Privacy Preserving Federated Blockchain Explainable Artificial Intelligence Optimization (PPFBXAIO) framework, which integrates blockchain technology, Explainable AI (XAI), and optimization techniques to ensure privacy, traceability, and robustness in FL-based systems. PPFBXAIO employs Secure Hash Algorithm 256 (SHA-256) for blockchain-backed secure model updates, Min-Max normalization for feature scaling, and the Levy Grasshopper Optimization Algorithm (LGOA) for optimal feature selection and federated model tuning. The Entropy Deep Belief Network (EDBN) is used as the classifier to enhance classification accuracy and detect attacks. XAI tools like SHAP are utilized to improve model interpretability. Experimental validation was conducted using the Heart Disease dataset from Kaggle and the Wisconsin Breast Cancer dataset. Results showed that PPFBXAIO achieved 95.07% accuracy, 95.44% precision, 96.54% recall, 95.98% F1 score, and reduced training loss by 4.93% for Breast Cancer Wisconsin and achieved 93.07% accuracy, 91.19% precision, 95.39% recall, 93.24% F1 score for Heart Disease dataset. Proposed system has reduced latency by 81 ms, and improved throughput by 109 transactions per second for 100 rounds as compared to traditional models like FedAvg, FL-MPC, FL-RAEC, and PEFL. These results highlight the framework’s superior performance, privacy preservation, and practical applicability in decentralized healthcare AI systems.

## Introduction

Progress in technology has led to the emergence of artificial intelligence (AI) educators, or more generally, automated systems for learning. AI has emerged as a disruptive force changing many aspects of technology, the economy, and society^1^. Ongoing advancements in computing power have significantly contributed to the expansion of machine learning, recognition of speech, and natural language processing, which are AI technologies^2^. AI-driven solutions are becoming increasingly significant in society and people’s lives^3^. Today, AI-based applications are prevalent across various domains, such as product recommendation systems^1^, smart cities, education, healthcare^4,5^, and autonomous vehicles. Unlike^1^, which integrates FL with blockchain primarily for secure model aggregation, our framework introduces an explainability layer that interprets model predictions at the client level and feeds explainability metrics into the optimization loop. Compared to^2^, which applies XAI post hoc to black-box healthcare models, our approach embeds XAI directly into the training pipeline within a privacy-preserving FL context, while also logging interpretability metadata on-chain for auditability. The novelty lies in the co-design of explainability and optimization with blockchain-enhanced FL in a unified framework tailored for healthcare constraints.

AI is progressively transforming medical practice, with numerous applications spanning various fields, including clinical care, diagnostics, rehabilitation, surgery, and predictive medicine. Conventional centralized AI models often lack transparency, exhibit reliability issues, and are vulnerable to data breaches. Clinical decision-making and disease diagnostics are other critical areas in which AI significantly impacts. To diagnose diseases, AI programs are capable of analyzing and understanding enormous quantities of data from numerous sources and assisting in making medical decisions^4,5^.

AI is transforming important fields where decision-making accuracy, privacy, and openness become increasingly challenging. However, centralized data processing threatens security and privacy infractions, as the conventional artificial intelligence models are black boxes without explainability. Although FL presents a distributed method of artificial intelligence training, it has trust and integrity problems. Among the interesting ideas used to handle the associated data privacy problem of centralized machine learning systems is FL. FL is distributed training at many nodes without passing actual data, guaranteeing data privacy but using collective intelligence instead^6,7^.

Utilizing a distributed machine learning method called FL, models may be trained together across several data sources without sending raw data. Unlike typical centralized methods, which gather data from many sources, FL divides the processing across local devices, or nodes, on a single server for training. Each device in the system transmits such weights or gradients to a central server and uses its private dataset for local model training. Combining these changes creates, for optimal performance, a server sends the global model to devices. This method improves data security while reducing the expenses and time required for processing large numbers^8^.

**Fig. 1:**
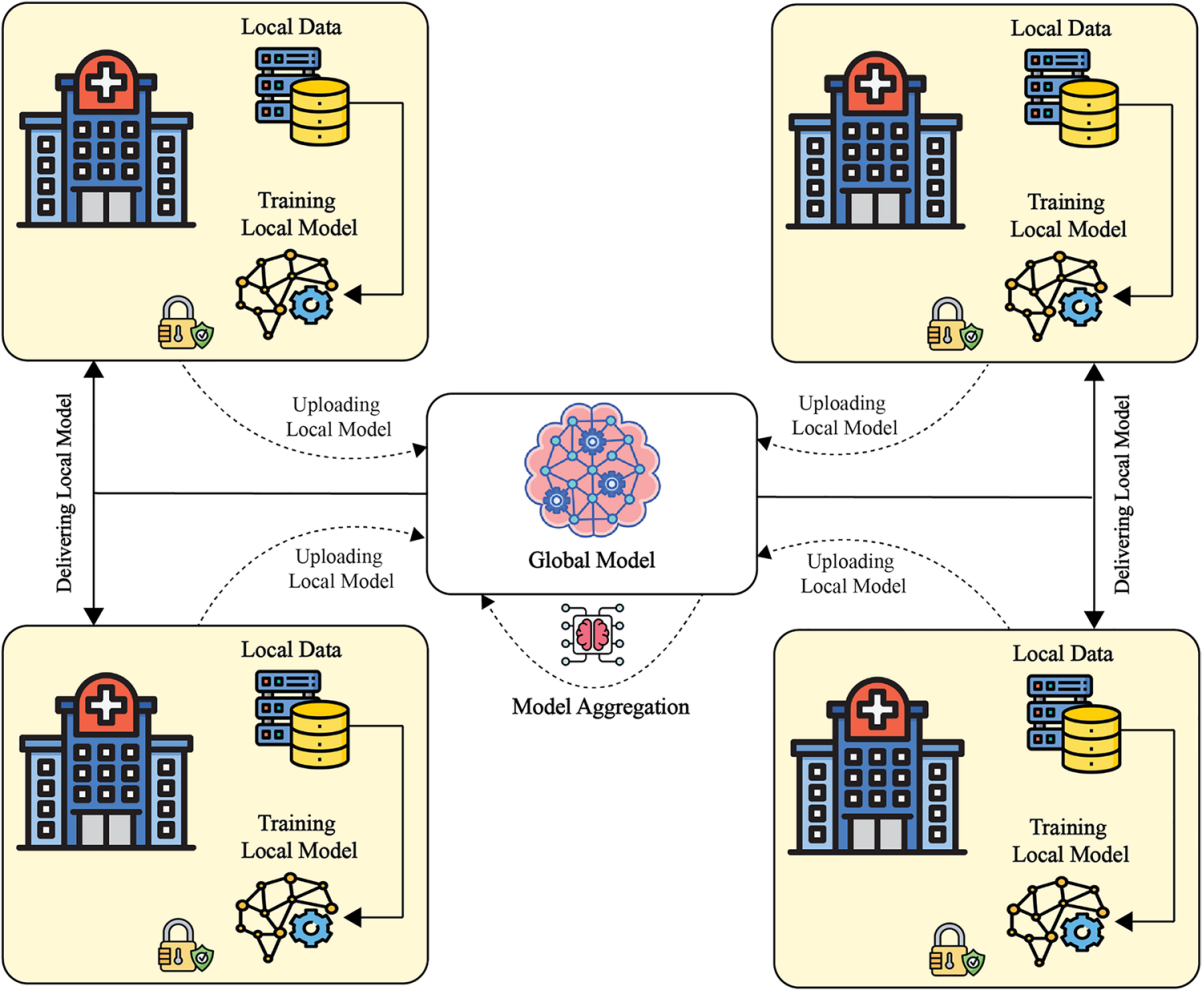
A FL Technique.

The FL architecture is seen in Figure 1, which prevents patient data breaches, strengthening intelligent healthcare systems. However, FL has major difficulties like vulnerability to single-point failures, sensitivity to poisoning attacks, and lack of strong incentive systems. Blockchain Technology (BCT) has been included in FL to assist with these problems using its distributed and unchangeable ledger to consistently, transparently, and securely store data^9^. Instead of forwarding updates to a central server, trainers give local model modifications to miners in blockchain-integrated FL for validation and global model aggregation. Every blockchain miner generates a global model independently, but only those satisfying certain requirements are qualified for release. Before being added to the blockchain, other miners verify the validity of the global model^10^. Blockchain-enabled FL offers several advantages for research. Firstly, Blockchain Technology (BCT) eliminates single-point failures, enhancing the training process’s resiliency. Secondly, malicious global models are largely rejected due to blockchain’s consensus mechanism, ensuring model integrity. Thirdly, all data records stored on the blockchain are tamper-proof, improving the dependability of the final aggregated model while preventing malevolent servers from compromising model training. As a result, BCT supports the core objectives of FL^10^. Traditional FL models, however, function as black boxes with limited transparency, making them unsuitable for critical fields such as healthcare^11^. FL and explainable AI (XAI) address decentralized training and interpretability, but they lack inherent mechanisms for enforcing data integrity, traceability, and trust in multi-institutional environments. Blockchain complements FL by providing an immutable ledger for model updates, securing communications between participating nodes, and managing access control through smart contracts. Without blockchain, the system remains vulnerable to tampering, rollback attacks, and unverified contributions. Optimization is essential to adaptively fine-tune model performance across heterogeneous nodes under privacy constraints, which cannot be achieved solely with FL and XAI.

XAI is crucial for addressing the ”black-box” nature of AI models. XAI approaches improve machine learning’s interpretability results by making the decision-making process explicit. This openness guarantees compliance with regulations and promotes stakeholder trust, especially in critical industrial applications^12^. XAI enhances interpretability in models, but existing models significantly lack consistency and transparency in explanations^13^. Blockchain has been suggested for security; however, XAI and the situation of FL in blockchain have hardly been explored yet. Considering these shortcomings, in this paper, the Privacy Preserving Federated Blockchain Explainable Artificial Intelligence Optimization (PPFBXAIO) framework is introduced to ensure secure data management, transparency, privacy, and a secure and efficient method through the integration of blockchain technology with FL, XAI, and optimization. The PPFBXAIO Framework has been introduced to promote trust, interpretability, and efficiency. PPFBXAIO are compared with typical FL schemes like FedAvg, FL with Multi-Party Computation (FL-MPC), Federated Learning with Robust Aggregation in Edge Computing (FL-RAEC), and a privacy-preserved and efficient FL framework with blockchain (PEFL), demonstrating better defense against various attack models.

The primary contribution is the design of a blockchain-enhanced federated learning framework where each participating node trains models locally on sensitive health data and generates explainable predictions using SHAP-based attribution. These interpretability summaries and model update metadata are cryptographically logged on a permissioned blockchain, ensuring tamper-proof traceability and auditability of decisions. This approach directly addresses the clinical demand for transparent AI, combining decentralized privacy-preserving training with immutable, explainable evidence to support trustworthy medical decision-making.

The PPFBXAIO system uses blockchain and federated learning to allow decentralized, tamper-proof model training across healthcare nodes without sending raw patient data. Its innovative optimization approach updates model weights securely via homomorphic encryption and differential privacy, protecting data confidentiality throughout aggregation. Blockchain smart contracts regulate model modifications and access, guaranteeing auditability and data integrity. Integrating explainable AI at the edge layer produces real-time, interpretable outputs utilizing SHAP-based feature attribution, enabling doctors to comprehend model choices locally without a central server. Existing healthcare AI systems lack safe distributed training, immutable logging, privacy-preserving optimization, and decentralized explainability.

## Literature review

Wang et al.^14^, presented Platform-Free Proof of Federated Learning (PF-PoFL), a novel energy-recycling consensus technique that accomplishes meaningful FL tasks by using the computing resources often squandered on solving difficult but ineffective Proof of Work (PoW) problems. However, security risks and efficiency challenges may arise because of the unreliable surroundings and the miners’ self-centered behavior. PF-PoFL’s innovative transaction types, credit-based incentives, and block structure system prevent spoofing and Sybil attacks, enabling the distribution of rewards, model validation, federated mining, and AI task outsourcing. Furthermore, PF-PoFL protects against implicit privacy leaks during FL model training by implementing a mechanism for miners to use differential privacy at the user level. A game-based approach for federation establishment is also introduced to account for dynamic miner characteristics across various FL tasks, enabling the decentralized construction of a Nash-stable, optimal, disjoint miner-divided structure. Extensive simulations confirm the efficacy and efficiency of PF-PoFL.

Singh et al.^15^ integrate blockchain with FL in smart healthcare architecture using blockchain-based IoT cloud platforms to protect data. Federated learning lets healthcare consumers connect to well-trained models without cloud data. The research also explored creating a dispersed, safe environment in smart cities using FL.

Djolev et al.^16^, presented aggregation techniques based on two FBLearn platform use cases: utilizing Logistic Regression (LR) to identify credit card fraud and using a Random Forest (RF) classifier to assess credit risk. Using blockchain smart contracts, the platform allows FL participants to securely store model data in decentralized storage (IPFS), communicate through a decentralized application (dApp) as the front end, and manage the development of the final model and interactions throughout data training. Utilizing validation data at either the local or global level for model assessment, techniques for aggregating global federated learning models that have been presented include either local model averaging or ensemble methods. The accuracy of the final global model is larger; so, the better performance of the proposed system over models trained on isolated datasets emphasizes its potential to boost cooperation and improve over individual local models. While maintaining anonymity, blockchain and FL provide a new data collaboration paradigm. Based on local training data quality, results of tests show that adaptive weight computation and ensemble procedures greatly increase global model endurance.

Gupta et al.^17^ presented a fresh approach to group model training with data privacy using Blockchain and FL technologies. Under this paradigm, FL allows on-device learning without exchanging raw data, while blockchain authenticates patient data. The framework classifies lung diseases using the DenseNet-201 model and the FedAvg technique to collect model parameters and securely store them on the blockchain via IPFS. Using Python and well-known libraries like TensorFlow and Scikit-Learn, a thorough assessment showed the algorithm’s efficacy in identifying lung disorders, attaining 90% accuracy, precision, recall, and F1-score. Dipto et al.^18^, the distributed identification of deformations in Red Blood Cell (RBC) images, suggested the difficulty of centralized gathering data. Training many Deep Learning (DL) models on RBC data helps one to choose the most effective model to act as the global model within the FL architecture. The FL technique averages global model weight updates after distributing its weights to local client models, training them on client-specific devices, thus improving the global model. Both weighted and direct averaging are included under the averaging process; the latter gives specific local models performance-based weights. This strategy protects the FL system from any assaults and the impact of unreliable customers. Client data stays private during training; the global model provides important insights. Finally, explainable artificial intelligence methods motivated by Grad-CAM verify the categorization findings to guarantee dependability and transparency.

Lohachab and Kumar^19^ presented FedHFP, a Federated Deep Learning model intended for heart failure prediction, stressing its relevance and adaptability in far-off places with inadequate medical resources. To improve heart failure prediction using FL, also carefully compared many deep learning models, including Long short-term memory (LSTM), recurrent neural networks (RNN), convolutional neural networks (CNN), and artificial neural networks (ANN), and gated recurrent units (GRU). With an accuracy of 93.75%, ANN may outperform other models according to a comparison study across many network configurations. Furthermore, this work theoretically investigates the relevance of these results in far-off locations with limited healthcare services. The findings highlight the efficiency of FL in predictive healthcare modeling as it can lower communication overhead and protect data privacy, therefore benefiting areas with inadequate healthcare infrastructure.

Wei et al.^20^ suggested DeFedHDP, a distributed online aggregation technique under fully decentralized federated learning (DFL), to address privacy concerns and improve the HDP concept. DeFedHDP also protects patient privacy by using the differential privacy (DP) mechanism in its aggregation method. Without a central server, the data bearer interacts directly with neighbors in a sequence of time-varying directed graphs. Moreover, every participant collaboratively works with the other participants’ models and the local model’s trainer. It simply requires combining and changing the model parameters; the data leaves the local device, so this distributed technique may help to increase the degree of privacy protection even further. Furthermore, the One-Point Bandit Feedback (OPBF) approach estimates actual gradient values to handle model gradient disappearance and explosion. Regarding accuracy and speed, studies using a publicly available medical dataset show that DeFedHDP’s performance is somewhat similar to the centralized FedAVG algorithm for client-server systems.

Otoum et al.^21^ proposed a new method integrating the TabNet model with the benefits of deep neural networks and tree-based models for the prediction of heart disease. Using the architecture of TabNet, the work employs the University of California (UCI) Heart Disease and Comprehensive Heart Disease datasets to improve data processing in federated environments. The federated averaging approach is used in horizontal FL to compile model updates across many users securely. Furthermore, incorporated to increase responsibility and openness is blockchain technology, which smart contracts help to provide automatic governance. With an epsilon value of 6.855 and an accuracy of 0.822, TabNet effectively maintained a balance between privacy and performance, according to experimental data, after 50 epochs, obtaining a highest balanced metrics score of 1.598. Additionally, using aggregated data in just 10 phases, the model showed excellent accuracy, highlighting the benefits of combining data from several sources. By integrating TabNet with blockchain, this study provides a private and scalable method for predicting heart disease, addressing important healthcare issues, and maintaining data integrity.

Khan et al.^22^ proposed improving the Iterative Artificial Bee Colony Algorithm (I-ABC) through FL to minimize data collecting node privacy and secrecy issues. This method will be beneficial in determining the important values of observations from the pool of data flowing across the data nodes or sensors, therefore predicting cardiac illnesses. With our iterative optimization approach, we achieve projected improvements in accuracy of the disease diagnosis, error reduction, and data economy. The result of the suggested FL is among the many modern approaches used in this context.

Asad and Otoum^23^, combining threshold Paillier encryption with threshold signature authentication and blockchain technology, produced a Blockchain-Based Framework for Privacy-Preserving Federated Learning (BPPFL). Secure participant identification is guaranteed by the BPPFL architecture, which also guards against external and internal threats. Blockchain also serves as an unchangeable record for transactions and model modifications, enhancing security and openness. While significantly reducing the communication and processing overhead compared to current techniques, BPPFL maintains exceptional model correctness and strong privacy safeguards. This design makes FL applications more reliable and safe, which qualifies them for the IoT, healthcare, and finance industries.

Liu et al.^24^ introduced the FL-RAEC, thereby preserving privacy. To protect Edge Server (ES) integrity and confidentiality, a hybrid privacy-preserving approach is designed. Second, a method of phased aggregation is used to improve model aggregation’s resilience. Third, anomaly detection uses an autoencoder; the initial step involves selecting individual ES units for anonymous trust assessment. Each ES’s trust score is then evaluated by many rounds of random verification, which also helps find malevolent participants. At last, the assessment of FL-RAEC shows its great dependability and precision under many assault situations.

Tian et al.^25^ demonstrated an efficient, privacy-preserving FL architecture with blockchain (PEFL). To coordinate privacy protection across clients, PEFL uses blockchain and differential privacy approaches; it also filters out aberrant model parameters using an aggregation-side detection method to prevent poisoning attempts. Balancing efficiency expectations to control the server and guarantee the dependability of the training process, the model-validated fault-tolerant federation (MFF) consensus method is based on a committee under the assumption of an untrusted server. PEFL shows improved security against certain attack models employing trials on the Modified National Institute of Standards and Technology (MNIST) and Canadian Institute for Advanced Research 10 (CIFAR10) datasets, and comparison with conventional FL schemes. Besides, it guarantees private security and produces better training efficiency.

Aitizaz Ali et al.^26^ suggested neural network-based secure searchable encryption for healthcare systems enabled by blockchain technology and industrial IoT. Blockchain is proposed as a homomorphic encryption distributed database in this research mechanism to provide safe access to the database via keywords and secure searches. The suggested method also appropriately updates different rules and offers a safe way to revoke keys. Therefore, blockchain technology and trust chains are used to create a secure patient healthcare data access system that addresses the efficiency and security issues of present digital healthcare data exchange schemes. As a result, our suggested method offers cost-effectiveness, increased efficiency, and transparency. Hyperledger Fabric, a blockchain-based technology, and OrigionLab, an assessment and analysis tool, were the foundations around which we built our simulations. The author contrasted the outcomes of the suggested models with those of the reference models. The comparisons clearly show that the suggested framework offers improved security and a searchable methodology for healthcare systems.

Mohammed Amin Almaiah et al.^27^ proposed Digital Healthcare IoT-Based CPS Hybrid Trustworthy Decentralized Authentication and Data Preservation Model. This study presents a method for decentralized authentication across legal devices that is both lightweight and secures data for Internet of Things (IoT) based CPS. The method makes use of deep learning (DL). Using decentralized authentication, in addition to improving communication statistics, we have decreased validation delays among collaborating devices. Additionally, to recognize the importance of our model, we compared the experimental findings with the benchmark models. The proposed model exhibits a significant improvement in relative parameters when compared to benchmark models.

Aitizaz Ali et al.^28^ discussed the Digital healthcare systems with blockchain-based security, privacy, and dependability. A permission blockchain is one way to make healthcare data sharing more secure and private. The literature indicates that centralized systems, which are more susceptible to security vulnerabilities, are used by the most of healthcare organizations. A platform for data interchange is offered by the existing healthcare blockchain schemes; however, these schemes do not address security and privacy issues. The author has developed a new security algorithm to address these types of threats; it offers privacy and security at a reduced cost and much improved efficiency. Therefore, in this research, we have introduced a patient healthcare framework that surpasses the state-of-the-art in blockchain-based access control regarding security, dependability, and authenticity. Aitizaz Ali et al.^29^ described Homomorphic Encryption-Based Privacy Protection in IoT Healthcare Applications: The HealthLock Blockchain. The study introduces a novel approach to enhance privacy protection in IoT-based healthcare applications by combining blockchain technology with homomorphic encryption techniques. Homomorphic encryption protects data privacy by analyzing encrypted data without decryption. Authorities may handle and analyze encrypted patient data without divulging its contents. To establish guidelines for data exchange and control who has access to what, the technique also makes use of blockchain smart contracts. Only authorized parties may decrypt and utilize the data thanks to the complex permission settings offered by these smart contracts. These settings make sure that the data is only accessible by authorized individuals. Additionally, our system maintains an audit history of every data transaction for increased transparency and accountability.

Aitizaz Ali et al.^30^ introduced the a Hybrid Deep Learning Model for Industrial IoMT Based on Consortium Blockchain and Homomorphic Encryption (HE). An essential contribution of this study is the integration of HE with the planned IIoMT system. Employing HE while storing data in the cloud offers an additional opportunity to do any machine learning or statistical analysis on the encrypted EMR data, making it resistant to phishing and collusion attempts. We provide a strategy that uses cloud-based pre-trained hybrid deep learning models and distributes them to blockchain-based edge devices for local model training and classification utilizing EMRs. That depends on the consortium blockchain’s security measures and the personal information of every edge and Internet of Things device using it. After each local model is collected, to update a global model, it is uploaded to the cloud. The updated model is then sent out to the edge nodes. Our suggested method outperforms traditional techniques in terms of privacy and security while still providing customers with great efficiency and minimal end-to-end latency. The author does simulated comparisons utilizing benchmark performance criteria to show that the recommended model improves upon state-of-the-art security, efficiency, and transparency. Nabeela Hasan and Mansaf Alam^31^ presented the Scalable System Architecture for Smart Cities Based on Cognitive IoT. Cognitive computing may assist in effectively managing massive amounts of data; however, there hasn’t been much good research in this area yet. Concerning data collecting in a smart city setting, scalability and adaptability are unsolved issues. In this chapter, the author look at the CIoT and suggest a smart city network that uses cognitive computing to solve scalability and flexibility issues. For the suggested design, we have also provided a variety of technologies, including AI and big data analysis. This domain’s potential and challenges are covered.

Nabeela Hasan et al.^32^discussed machine learning-based blockchain federated safety-as-a-service system for industrial IoT. The Blockchain Driven Cyber-Physical System (BDCPS) is supported by cloud computing and the IoT. Using a small-scale, real-world Blockchain connected to the Internet of Things (IoT), BDCPS will validate the assertion using Intelligent Agreements and a trustless, peer-to-peer, centrally managed database. Based on the results of this research, smart gadgets may operate in tandem with a private blockchain housed on its board system. Shipping, evaporation, lightweight, and warehousing transactions time are some of the problems the proposed solution uses blockchain technology to solve. Blockchain data flows demonstrate how machine learning may improve food traceability.Finally, a supply chain increases shelf life with correct data.

Nabeela Hasan et al^33^ deliberated early industry machine failure detection using unsupervised machine learning. The article explains the study’s trustworthy and prognostic analysis for machine failure detection in the industry. The dataset is used to train an interpretable technique and an informative functionality, which are then contrasted and assessed for explicatory implementation. This study develops a deployable end-to-end grading model to anticipate machine failure. Train and compare state-of-the-art gradient boosted decision trees (GBDT) methods.

Prior work on FL-XAI-blockchain integrations has either ignored the unique confluence of explainability, data privacy, and model auditability or has provided merely a surface level of connectivity between the various components. For instance, research using FL and blockchain often disregards tamper-proof validation and decentralized orchestration in favor of an emphasis on safe weight aggregation; research utilizing XAI in healthcare, on the other hand, heavily emphasizes transparency. Also, no current solution uses the feedback loop that would allow explainability insights to verifiably anchor in a blockchain ledger or immediately impact federated optimization. These omissions constitute important gaps in relevant research to enable responsible, privacy-preserving, and interpretable AI in regulated areas like healthcare. PPFBXAIO bridges this gap by integrating federated model governance, blockchain-based verification, and XAI-driven local explanations into a safe, auditable, and cohesive learning architecture.

## Proposed methodology

**Fig. 2:**
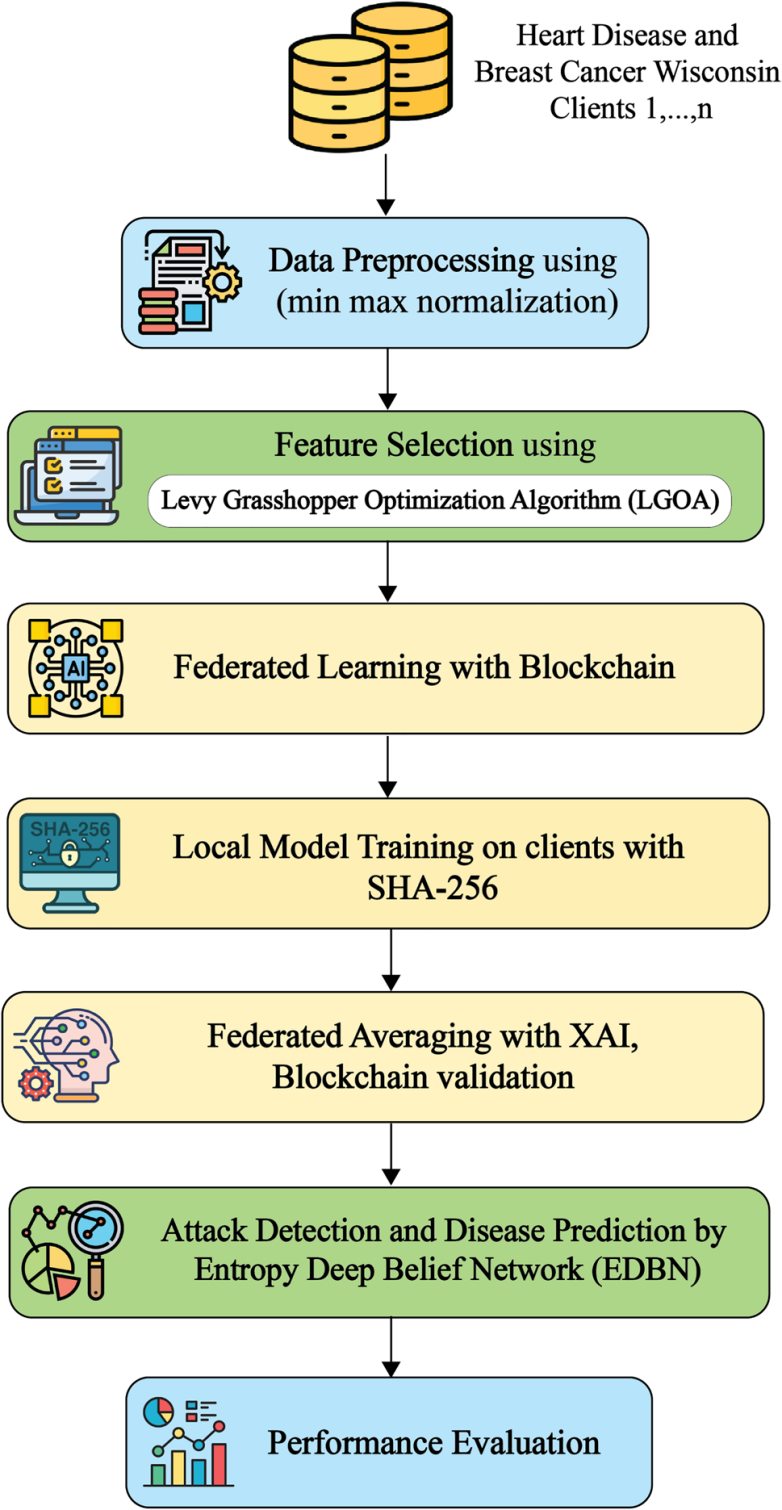
Proposed Framework For Blockchain-Assisted FL Workflow.

In this paper, the Privacy Preserving Federated Blockchain Explainable Artificial Intelligence Optimization (PPFBXAIO) Framework is introduced to ensure secure data management, transparency, privacy, and a secure and efficient method through blockchain technology’s incorporation with FL,XAI with optimization. PPFBXAIO combines blockchain and privacy techniques to coordinate privacy protection among clients and filters out anomalous model parameters through an aggregation algorithm to resist poisoning attacks. Data privacy is maintained while user participation can be improved by using FL and integrating AI models on consumer devices. Blockchain technology improves healthcare data management’s security and transparency, while XAI improves the interpretability of model decisions. Additionally,LGOA is introduced to XAI select the dataset’s most significant attributes, and it can optimize the hyperparameters of federated averaging with increased performance. Finally, classification and attack detection have been performed by the EDBN. The high-level design of the PPFBXAIO framework is given in Figure 2. One core focus is interpreting FL model predictions locally at each node using SHAP-based methods. These interpretations allow domain experts to validate model reasoning for individual predictions without accessing centralized data. The system uses feature attribution scores and cross-node explanation consistency to inform global model updates. This feedback loop guides the optimization layer in adjusting hyperparameters, aggregation weights, and personalization strategies to preserve both accuracy and interpretability across non-IID data distributions. While the blockchain primarily uses secure logging and access control, the framework records XAI metadata such as dominant features and confidence metrics on-chain. This enables audit trails for clinical decisions, allowing stakeholders to verify what decision was made, why it was made, and under which model version and data context.

### Dataset collection

In this research, the Heart Disease and Breast Cancer Wisconsin datasets have been collected from Kaggle to simulate a distributed environment in which each client has a unique dataset. The Cleveland, Hungary, Switzerland, and Long Beach V databases comprise the heart disease dataset, which may be accessed at https://www.kaggle.com/datasets/johnsmith88/heart-disease-dataset. Most of the research uses a subset of 14 of its 76 features. Heart disease is indicated by the ”target” variable, where 0 denotes the absence of the condition and 1 denotes its existence. In addition, characteristics from digital images of fine needle aspirate (FNA) samples of breast masses may be obtained in the Breast Cancer Wisconsin dataset, which can be obtained at https://www.kaggle.com/datasets/uciml/breast-cancer-wisconsin-data. They provide details on the features of the cell nuclei shown in the image. The datasets were prepared to reflect classification problems intended to predict binary outcomes based on multiple input features. The highly precise features from each dataset indicate critical factors relevant to diagnosing and treating different diseases, leading to research value in risk prediction and treatment strategy development. Class labels were assigned in terms of the linear combination of features after applying a sigmoid function to enforce non-linearity in the class assignments, resulting in hard classification tasks. Explainable AI Optimization in this framework refers to integrating interpretability feedback into the model optimization process within a federated learning environment. Instead of treating explainability as a post hoc analysis, the system captures local interpretability outputs such as SHAP or LIME-based feature importance and uses these as additional signals to influence model aggregation, personalization, and hyperparameter tuning. This creates a feedback-driven optimization loop where models are optimized for performance metrics (e.g., accuracy, loss), consistency, and transparency in their explanations across nodes.

### Data preprocessing using min-max normalization

Min-max normalization is used as a preprocessing method in classification to fit the raw dataset within a predetermined range, frequently between 0 and 1. Each feature’s smallest and maximum values are ascertained in this method, and all data points are adjusted proportionately. The smallest value is mapped to 0, the largest to 1, and all other values are scaled accordingly based on their relative position in the original dataset. The normalized value $x'$ is calculated using Equation (1),

1 $$\begin{aligned} x' = \frac{x - \min (x)}{\max (x) - \min (x)} \end{aligned}$$

where *x* represents the initial value, the feature’s smallest value is $\min (x)$, and its highest value is $\max (x)$.

### Feature selection using Levy Grasshopper Optimization Algorithm (LGOA)

Feature selection has been recognized as an effective and efficient method for preparing datasets in disease prediction. Its primary objectives include creating simpler, more interpretable models, enhancing data-mining performance, and ensuring clean, well-structured data. Recently, meta-heuristic optimization algorithms have been widely utilized to identify the most relevant features. Typically, feature selection aims to decrease the feature space while preserving excellent predictive performance, considering it a multi-objective optimization problem. Swarming and foraging inspired LGOA grasshoppers to select features from a dataset. The grasshopper’s life cycle consists of two stages: nymph and adulthood. Small steps characterize nymph movements; in adulthood, movements become abrupt and cover long distances. These two phases represent the intensification and diversification stages of LGOA. Within the algorithm, each grasshopper symbolizes a potential feature selection solution in the population. The following method is used to resemble grasshopper swarming behavior and calculate each solution’s position Xi^34^,

2 $$\begin{aligned} X_i = S_i + G_i + A_i \end{aligned}$$

Where $X_i$ represented the selected feature solution among the other grasshopper swarms, $G_i$ represented the gravitational force acting on the selected feature solution, $A_i$ represented wind advection, and $S_i$ represented the interaction of the *i*-th grasshopper position for feature selection, as shown in the following equations (3-4)^34^.

3 $$\begin{aligned} S_i= & \sum _{j=1}^{N} s(d_{ij}) \hat{d}_{ij}, \quad \text {where } j \ne i \end{aligned}$$

4 $$\begin{aligned} s= & f e^{-r/l} e^{-r} \end{aligned}$$

where *N* stands for the number of grasshoppers, $d_{ij} = |x_j - x_i|$ is denoted as the grasshoppers’ swarm’s *i*th and *j*th Euclidean distances, $\hat{d}_{ij} = \frac{|x_j - x_i|}{d_{ij}}$ is represented as the grasshopper swarm’s unit vector from the *i*th to the *j*th. Additionally, *l* is the attractive length scale, attraction and repulsion between grasshopper swarms are two social factors, with *f* representing the level of attraction and *s* representing their strength. To get the force of gravity $G_i$, use equation (5) below^34^.

5 $$\begin{aligned} G_i = -g \hat{e}_{g} \end{aligned}$$

where $\hat{e}_{g}$ is the unit vector and *g* is gravity. The equation (6) shows how to compute $A_i$^34^.

6 $$\begin{aligned} A_i = u \hat{e}_{w} \end{aligned}$$

Where $\hat{e}_w$ is the wind direction unit vector, and *u* is the drift constant. Equations (3-6) may be used to restore Equation (7) when the values of $S_i$, $G_i$, and $A_i$ have been reconstructed[26].

7 $$\begin{aligned} X_i = \sum _{j=1}^{N} s(d_{ij}) \hat{d}_{ij} - g \hat{e}_{g} + u \hat{e}_{w} \end{aligned}$$

However, the feature selection issue and the inability to converge at the area of the grasshopper swarms objective cannot be resolved simply using the mathematical equation model (7). It has been described as follows^34^.

8 $$\begin{aligned} X_i^d = c \left( \sum _{j=1}^{N} c \frac{(UB_d - LB_d)}{2} s(|x_j^d - x_i^d|) \frac{|x_j - x_i|}{d_{ij}} \right) + \hat{T}_d \end{aligned}$$

Where $UB_d$ and $LB_d$ are the d-th feature dimension’s upper and lower boundaries, respectively, and $\hat{T}_d$ represents the d-th dimension space’s best feature solution to date. In Equation (8), there is no $G_i$ component, thus the force of gravity is not taken into account. Consider that the wind’s direction ($A_i$) is always the same, in the direction of the target $T_d$. The second component simulates grasshoppers’ propensity to approach the food source, $\hat{T}_d$^34^

Equation (8)’s parameter *c* is essential to the LGOA algorithm’s feature selection process since it balances local and global search. The inner *c* within Equation (8) helps decrease repulsion and attraction, and the comfort zone among grasshopper’s iterations increases. Equation (8)’s outer *c* regulates the movement of grasshoppers around the target (food), gradually narrowing the search space as iterations increase. The coefficient *c* has the following definition^34^.

9 $$\begin{aligned} c = c_{\max } - t \left( \frac{c_{\max } - c_{\min }}{t_{\max }} \right) \end{aligned}$$

Here, $c_{\max } = 1$ and $c_{\min } = 0.00001$ represent the maximum number of iterations and the current iteration (*t*) ($t_{\max }$), respectively, whereas *t* and *c* represent the extremes of *c*, both minimum and maximum. A grasshopper’s present location, the globally optimum feature position, and other grasshoppers in the swarm showing different feature placements are all taken into consideration while updating its position. The following is an approximation of the Lévy flight step variation probability density function^34^.

10 $$\begin{aligned} L(s) \sim |s|^{-1-\theta } \end{aligned}$$

Here, *s* represents the Lévy flying behavior’s random step length, while $\theta$ is constrained within the range [0, 2] as a power-law index. In the Lévy distribution graph, it is usually set to 1.5, which affects the sharpness of the peak. Varying the parameter $\theta$ results in different distribution patterns. The random walk step length *S* in Mantegna’s three-step technique is ascertained as follows^34^.

11 $$\begin{aligned} S = \frac{U}{|V|^{1/\theta }} \end{aligned}$$

Where *U* and *V* are normal stochastic variables with standard deviations of $\sigma _U$ and $\sigma _V$, respectively, whereas *S* is the variable for the random step length. Normal distributions should be used to determine *U* and *V*^34^.

12 $$\begin{aligned} U \sim N(0, \sigma _U^2), \quad V \sim (0, \sigma _V^2) \end{aligned}$$

The symbol $\sim$ in Equation (12) indicates that distribution samples are taken, as the random variable follows the distribution specified on the right-hand side. Since individual selection of standard deviations $\sigma _U$ and $\sigma _V$ is not possible for any given $\theta$ value, they are often set to the same value, as described in^34^.

13 $$\begin{aligned} \sigma _V = 1 \end{aligned}$$

Equation (14) provides the standard deviation $\sigma _V$ after this design^34^.

14 $$\begin{aligned} \sigma _U = \left( \frac{\Gamma (1+\theta ) \times \sin (0.5\pi \theta )}{\Gamma \left( 0.5(1+\theta )\right) \times \theta \times 2^{0.5(\theta -1)}} \right) \end{aligned}$$

The step size in Lévy flight is determined using equations (10-14), which model both short-distance searches and occasional long-distance movements. Following that, we use the following equation (15) to determine the step size.

15 $$\begin{aligned} \text {stepsize} = f \times S \end{aligned}$$

In this context, the factor *f* ($f = 0.01$), derived from *L*/100, controls the Lévy walk, where *L* is for the Lévy flights and relies on the problem’s feature dimension. This factor guides new feature solutions excluded from the existing search region. The step size value is incorporated into the update equations (10-14) of the LGOA algorithm to determine the optimal feature position. An effective mathematical operator that increases the number of feature solutions in the search space and improves the exploration capability of the LGOA algorithm is the Lévy flight distribution.

All grasshoppers, except the first one, which is assigned random values, are initialized using Lévy flight distribution values. This approach ensures a more diverse starting point, enhancing the initialization phase. Furthermore, during the iteration process, the Lévy flight mechanism helps the algorithm effectively reach the target by overcoming limitations, avoiding local optima, and restarting in different regions of the feature search space. Algorithm 1 describes the LGOA algorithm’s pseudo-code.

**Algorithm 1:**
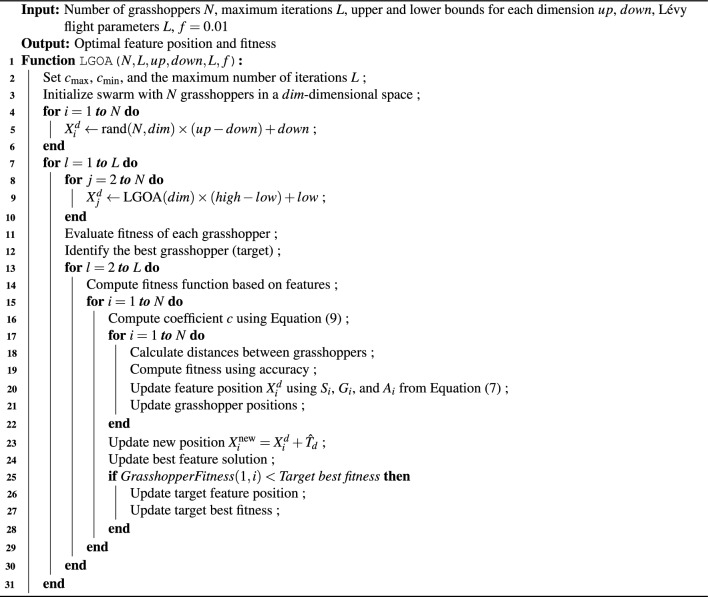
Lévy Grasshopper Optimization Algorithm (LGOA)

### Privacy-Preserving Federated Blockchain Explainable Artificial Intelligence Optimization (PPFBXAIO)

The specifics of the FBXIAO framework are covered in this section. By using AI models on FL-enabled consumer devices, data privacy is maintained while user collaboration in learning is promoted. Blockchain technology improves healthcare data management’s security and transparency, whereas XAI improves the interpretability of model decisions. Additionally, the LGOA is introduced to XAI select the dataset’s most important characteristics, and it can optimize the hyperparameters of federated averaging with increased model performance.

#### Federated learning

The Flower architecture enables decentralized training across several client devices using the FL process^35^. The server adds each client’s local model weights to the training using a private dataset to generate a global model. The method of dispersed instruction is very beneficial in the medical field. Multiple clients, which may be devices or organizations, can work together to train a model using FL, a decentralized machine learning paradigm, without transmitting raw data. This guarantees confidentiality and privacy because the data is localized on client devices and only model modifications are shared. Local client-side training is the first step in the process. Individual clients train private data models. A Flower server is the central coordinator for model updates once all locally trained models have been combined. A central server and multiple companies are often involved in FL. Participants train the shared models and then gather and redistribute them to the server. In FL, the training procedure is usually divided into three main steps^36^,

1. **Step 1: Task initialization-** The server selects the devices that will participate in FL establishes the tasks and objectives, and selected recipient receives shared model clients before training begins.
2. **Step 2: Local training and updates-** Each device uses a private dataset to train a local model, aiming to optimize its performance. Once training is complete, the model parameters are uploaded to the server for the subsequent phase.
3. **Global aggregation and download-** The server gathers model parameters from all participants and combines them through an averaging process to generate the revised global model for the next training session. The objective is to refine the global model for optimal performance. Once the new version is derived, all clients can access the server’s updated global model (wG) to improve their local models in the following iteration. The FL process continues until the worldwide function of loss attains stability or achieves the requisite accuracy. However, communication efficiency and security while transmitting local updates remain significant concerns. Malicious participants may introduce faulty training samples or compromised models, leading to failures in machine learning predictions. Additionally, unauthorized users can manipulate or corrupt stored models, threatening the system’s integrity.

**Fig. 3:**
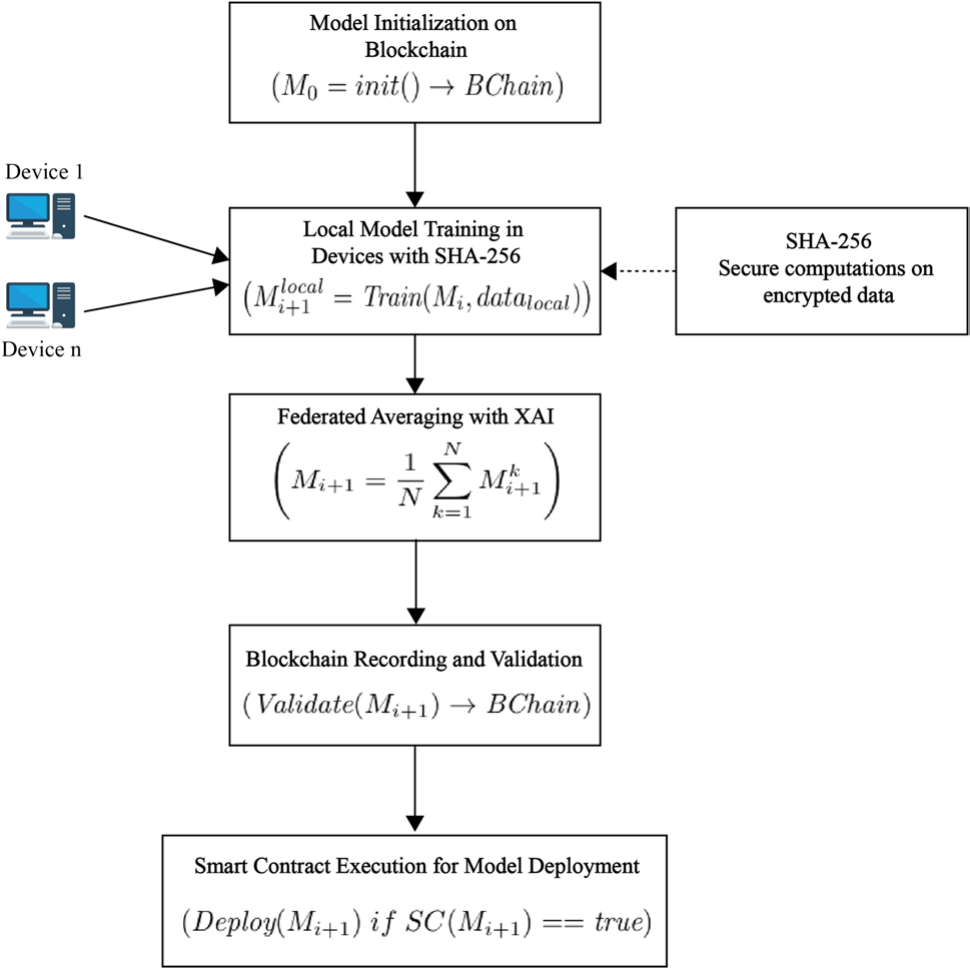
Workflow of Proposed Framework for PPFBXAIO.

The framework’s workflow is shown in Figure 3, which details each stage of the procedure. The model must first be initialized to preserve transparency and integrity, and basic parameters are securely saved on the blockchain. Computation on encrypted data is then made possible by local model training that occurs on individual devices using SHA-256. XAI is used to evaluate feature importance using LGOA, improving the global model, and the device updates are then aggregated using federated averaging. During the blockchain recording and validation phase, the aggregated model undergoes verification and is securely stored on the blockchain, ensuring that only authorized updates are incorporated. Lastly, if the verified model satisfies certain quality standards, smart contracts enable the automated distribution of the model to devices.

#### Model initialization on blockchain

The first machine learning model parameters are securely maintained during the model initialization process, and they are designated as $M_0$, on the blockchain, establishing the groundwork for collaborative learning. This process is defined as $M_0 = \text {init}() \rightarrow \text {BChain}$, where the model parameters may be safely stored on the blockchain. By placing the model’s original documentation on the blockchain, the architecture ensures a clear and unchangeable record of its evolution, strengthening security and confidence in the cooperative learning environment. The foundation of training in a federated network is this initialization. Storing $M_0$ on the blockchain ensures a validated starting point, promoting accountability and preventing unauthorized alterations. This technique maintains decentralized trust and data integrity throughout the learning process.

Data access and modifications in FL settings are transparent and secure using blockchain technology. Blockchain integration ensures the integrity and immutability of the results, creating a traceable and auditable record of the FL process. This FL, optimization, explainability, and blockchain combination builds a privacy-preserving, secure, and transparent learning ecosystem. The Blockchain Service also plays a major role in being transparent and accountable. It initiates with the genesis block, which initializes the blockchain. As it trains, the block’s values, predictions, and other metadata are added to the blockchain. Each block will be validated with the cryptographic hash value to ensure the data is not manipulated. Blockchain ensures the entire training process and prediction are tamper-proof, creating confidence in the FL architecture. With the blockchain, the transparency of an audit trail can be enabled, making stakeholders trace the evolution of the model and verify its reliability.

#### Training local models on devices

The next stage is local model training using each device’s data. In this approach, $M_{i+1}^{\text {local}} = \text {Train}(M_i, \text {localdata})$, where $M_i$ represents device-specific local data and global model parameters at iteration *i*. This method reduces the requirement for raw data, protecting user privacy while enabling devices to train models independently. Each device may use its data features thanks to local training, improving model adaptability. Conducting training locally allows the framework to leverage diverse data sources while maintaining privacy, aligning with the fundamental goal of federated learning (FL) data security and a decentralized training model.

The Secure Hash Algorithm 256 (SHA-256) cryptographic algorithm hashes any input data to 256 bits. The encrypted data retains its original length or may vary in size, depending on the encryption method used. In contrast, hashing converts data into an output of a set length. Unlike encryption, it is difficult to determine the original material from its hash value since hashing is a one-way operation. It would take $2^{256}$ tries to recreate the original data in a brute-force assault. Furthermore, there is very minimal chance that two distinct inputs would result in the same hash value, a situation known as a collision. With $2^{256}$ possible hash values far exceeding the number of atoms in the observable universe, the probability of two identical hashes occurring is extraordinarily low. Moreover, slight modifications to the original data produce a significant difference in the hash value, making it nearly impossible to recognize any similarity between the original and altered data. This phenomenon is known as the avalanche effect.

In the given code, SHA-256 is used on the blockchain to provide each data unit a unique hash. Hash function inputs map to fixed-length outputs H(x),

16 $$\begin{aligned} H(x) = \text {SHA-256}(x) \end{aligned}$$

Here, *x* is the block data, including its index, timestamp, content, and the hash of the previous block. This one-way function ensures that even a slight change in *x* produces a completely different hash *H*(*x*), making it collision-resistant. The compute hash function encodes a block’s data into JSON format, sorts its keys to maintain order, and applies the SHA-256 algorithm.

17 $$\begin{aligned} \text {Hashblock} = \text {SHA-256}(\text {JSON}(\text {block})) \end{aligned}$$

This ensures the uniqueness and immutability of each block’s hash, safeguarding data integrity across the blockchain. Unlike encryption, hashing cannot be reversed, thus providing integrity rather than confidentiality. For aggregation, instead of standard averaging (FedAvg), we implement the proposed Explainability-Guided Aggregation (EGA) method, which weights client updates based on the alignment of their local explanation profiles with a trusted global explanation reference. This prioritizes accurate and interpretable contributions, enhancing model robustness and transparency. This study also benchmarked our aggregation against FedAvg and FL-RAEC (Federated Learning with Robust Aggregation and Explainability Constraints), demonstrating that PPFBXAIO achieves higher accuracy and better interpretability metrics while maintaining competitive communication costs. The inference phase follows standard federated protocols where, for local prediction, clients get the global model.

#### Privacy preserving model

A local differential privacy (LDP)-based adaptive security-preserving technique is given to safeguard healthcare data privacy while improving data efficiency. The *i*th private data $x_i$ in the private source *X* is used to implement Randomized Aggregate Privacy-Preserving Ordinal Response (RAPPOR). *X* is a binary vector of length *k* such that $x[i] = 1$ and $x[j] = 0$ for $j \ne i$ $(1 \le i, j \le k)$, and $x[i] \in \{0, 1\}$ indicates the *i*th bit of *x*. That is, just the *i*th bit is set to 1 for the private data $x_i$ in *X*, while all other bits are set to 0. Keep in mind that each data owner performs the encoding process locally.

Let *y* be the result of *x*, and let $[i] \in \{0, 1\}$ represent *y*’s *i*th bit. A $2 \times 2$ conditional probability matrix $\Pr \{y[i] | x[i]\}$ that translates *x*[*i*] to *y*[*i*] with $\Pr \{y[i] | x[i]\}$ $(1 \le i \le k)$ describes the privatization mechanism based on simple RAPPOR for each bit of the encoded string *x*.

Equation (18-19) states that each data owner individually modifies the *i*th bit of *x*.

18 $$\begin{aligned} & \Pr \{y[i] | x[i]\} = {\left\{ \begin{array}{ll} p = \frac{e^{\epsilon /2}}{1+e^{\epsilon /2}}, & \text {if } x[i] = y[i] \\ q = \frac{1}{1+e^{\epsilon /2}}, & \text {if } x[i] \ne y[i] \end{array}\right. } \end{aligned}$$

19 $$\begin{aligned} & \text {which satisfies } \epsilon \text {-LDP, where } \epsilon = 2 | \ln (p/q) | \end{aligned}$$

Each bit of *x* in this instance has an expectation of *p* of keeping its value and a probability of *q* of changing ($p + q = 1$). Below is a theoretical derivation of the MSE for the estimate based on basic RAPPOR. Let *n* be the total number of owners of the data. Based on the maximum likelihood estimate (MLE) utilizing basic RAPPOR, the empirical estimation $\hat{P}_i$ of the true probability $P_i$ is obtained as follows:

20 $$\begin{aligned} \hat{P}_i = \frac{e^{\epsilon /2} + 1}{e^{\epsilon /2} - 1} \times \frac{n y_i^{bR}}{n} - \frac{1}{e^{\epsilon /2} - 1} \end{aligned}$$

where $n y_i^{bR}$ represents the value of 1 in the *i*th bit of the perturbed bit strings that are produced. Based on basic RAPPOR, the variance of the estimate $\hat{P}_i$ is obtained as follows:

21 $$\begin{aligned} \text {Var}[\hat{P}_i - P_i] = \frac{e^{\epsilon /2}}{n(e^{\epsilon /2} - 1)^2} + \frac{P_i}{n} - \frac{P_i^2}{n} \end{aligned}$$

Equation (22) then provides the MSE of $\hat{P}_X$ based on simple RAPPOR:

22 $$\begin{aligned} \text {MSE}(\hat{P}_X)_{bR} = \sum _{i=1}^{k} \text {Var}[\hat{P}_i - P_i] \end{aligned}$$

**Attacker model: ** In FL involving multiple parties, multiple malicious participants may collaborate to launch attacks, also known as collusion attacks. In this attack scenario, two or more malicious participants may privately collaborate to obtain the privacy information of other members or attack the global model. Let us assume the attacker controls $f-1$ devices or *f* participants to conduct collusion attacks. Specifically, attackers have knowledge of the code, local training datasets, and local models on $f-1$ devices. In particular, attackers can know the aggregation rules in various scenarios. Blockchain in PPFBXAIO ensures verifiability of model updates, enforces role-based access control through smart contracts, and logs hash values of model states and interpretability metadata for tamper-proof traceability. Unlike^1^, which uses blockchain for basic parameter exchange, our approach enables historical audits of the model’s learning trajectory and the rationale behind predictions, enhancing trust and regulatory compliance in healthcare applications.

#### Federated averaging with XAI integration

The aggregation procedure does more than add together model parameters; it simplifies federated averaging for improved comprehension. Instead, weighting criteria based on hyperparameters are utilized to evaluate data samples from each device. Since each local model influences the global model, devices with greater data sets have a stronger effect. The aggregation process follows the equation below:

23 $$\begin{aligned} M_{(i+1)} = \sum _{k=1}^N \frac{n_k}{n} M_{(i+1)}^k \end{aligned}$$

where $n = \sum _{k=1}^N n_k$ indicates the total dataset size across all participating devices, and $n_k$ indicates the amount of data samples on device *k*. This weighted approach improves the global model’s resilience and generalizability by preventing devices with smaller datasets from excessively impacting it. It also takes into consideration any changes to hyperparameters, such as local training epochs and learning rates, to further optimize each device’s contribution to the overall model.

In Equation (23), the weights $\frac{n_k}{n}$ are assigned $n = \sum _{k=1}^N n_k$, determined by the quantity of data samples $n_k$ on each device. In accordance with their statistical significance, our method guarantees that devices with larger data sets make a more substantial contribution to the global model. Additional aspects that may impact the weighting include device dependability (e.g., historical consistency), model convergence (e.g., reduced loss or greater accuracy), and data quality (e.g., completeness and reliability). The following is an expression for a generalized weighting function $w_k$:

24 $$\begin{aligned} w_k = \alpha \cdot \frac{n_k}{n} + \beta \cdot q_k + \gamma \cdot r_k \end{aligned}$$

In this formulation, $r_k$ represents dependability, $q_k$ represents data quality, while $\alpha$, $\beta$, and $\gamma$ represent hyperparameters. The aggregation process may consider input the amount and quality, using this flexible method, promoting equity and improving the FL framework’s resilience.

The system uses Explainable AI (XAI) approaches to enhance aggregation. After aggregation, it generates interpretations like gradients and attention maps to assess the importance of various data sources and highlight important characteristics. As shown below, this data-driven strategy allows the system to prioritize high-impact data and improve model aggregation.

1. To maximize the effect of the FL process, the framework’s crucial characteristics are enhanced by including XAI. XAI techniques help determine feature significance and guide prioritization, especially for sensor inputs susceptible to errors. This adaptive mechanism enhances model resilience across diverse operational scenarios, improving interpretability and reliability.
2. Local model contributions vary depending on its assessed relevance to reinforce the insights obtained via XAI. After update cycle $i+1$, weight device *k*’s model update, represented by $W_{i+1}^k$. The following is the formulation of the modified aggregation process:
  25 $$\begin{aligned} M_{i+1} = \frac{\sum _{k=1}^N W_{i+1}^k M_{i+1}^k}{\sum _{k=1}^N W_{i+1}^k} \end{aligned}$$
  Using XAI insights, by selecting data sources that provide more accurate and dependable forecasts, this technique improves the global model.
3. XAI further customizes the training procedure for every device during local training by giving data points related to earlier model faults priority. A significant score $\alpha _t^k$ is used to modify the local loss function $L^k(M^k, x_t^k)$.
  26 $$\begin{aligned} L_{\text {XAI}}^k (M^k, x_t^k) = \alpha _t^k \cdot L^k (M^k, x_t^k) \end{aligned}$$
  Prioritisation improves learning and reduces repeated errors by assisting the model in adapting to difficult data points.
4. The framework handles situations by using a prototype and criticism process. The model is able to concentrate on situations that XAI deems troublesome or archetypal by using the loss function to emphasize such data. In equation (27) this weighted function is provided,
  27 $$\begin{aligned} L_{\text {proto-crit}}^k = \beta L^k (M^k, x_{\text {proto}}) + \gamma L^k (M^k, x_{\text {crit}}) \end{aligned}$$

The XAI framework integration generates A self-optimizing feedback loop, constantly improving the model in response to real-time data. The following illustrates this iterative process:

28 $$\begin{aligned} M_{i+1} = \frac{\sum _{k=1}^N W_{i+1}^k \left( M_{i+1}^k + \Delta M_{\text {XAI}}^k \right) }{\sum _{k=1}^N W_{i+1}^k} \end{aligned}$$

This includes modifications based on XAI, which increase the model’s security and resilience. In the PPFBXAIO Framework, the XAI component integrates three key techniques to generate and utilize model interpretability: SHAP (SHapley Additive exPlanations), LIME (Local Interpretable Model-Agnostic Explanations), and Grad-CAM (Gradient-weighted Class Activation Mapping). In the PPFBXAIO Framework, model updates are validated and stored through a two-tiered mechanism combining blockchain consensus and explainability-aware validation.

#### Blockchain validation and smart contract deployment

The global model is validated following an update to ensure it exceeds predetermined accuracy standards. This procedure ensures that only model updates of the highest quality are considered for deployment. The blockchain provides an unchangeable and validated model to create an open record of each model version $M_{i+1}$ as $\text {Validate}(M_{i+1}) \rightarrow \text {BChain}$. The framework provides a strong method to preserve confidence in the FL procedure by maintaining data accuracy and preventing unwanted alterations by only recording approved updates. Before implementing the enhanced model, the framework assesses its correctness using smart contracts. These contracts ensure that only models that satisfy certain performance requirements are deployed by imposing predetermined constraints, which are stated as $\text {Deploy}(M_{i+1})$ if $\text {SC}(M_{i+1}) = \text {true}$.

A self-executing software on the blockchain is identified as a smart contract, capable of carrying out anything from validating aggregated model updates and storing results to actions further down based on rules already set. No middlemen exist in a smart contract, and the promise of faster, more reliable operations comes with that. Smart contracts validate updates using this methodology before adding them to the distributed ledger. An example is if the contract will first verify if aggregated updates achieve a minimum acceptable level of accuracy, having been properly computed. Logging only reliable data into it ensures the training procedure is sound. Smart contracts can also be set up to trigger some actions. Hash functions and consensus mechanisms make the data tamper-proof since nobody can play any game with the data. A series of blocks serves as the ledger’s representation,

29 $$\begin{aligned} B = \{b_1, b_2, \ldots , b_n\}, \quad i = 1, \ldots , n \end{aligned}$$

where $b_i$ is represented as the *i*-th block. A hash function is used for cryptographically linking each block. This function is represented as follows:

30 $$\begin{aligned} H(b_i) = \text {Hash}(b_{i-1} + \text {Data}) \end{aligned}$$

where *H* is the hash function ensuring data integrity. Updates are validated so that the validity of the blockchain can be maintained.In FL, after aggregating the updates from multiple clients, an international validation dataset assesses the model’s performance (accuracy).This ensures that the aggregated model performs well across different data distributions. To execute the smart contract, equation (31) is given as below:

31 $$\begin{aligned} \text {SC}(f_i) \Rightarrow \text {Execute action if condition } f_i \text { is satisfied.} \end{aligned}$$

SC indicates smart contract, and $f_i$ indicates condition ($\text {accuracy}> \text {threshold}$). The validation condition ensures that model updates meet performance standards before being accepted in the distributed ledger. Data integrity and immutability are ensured using hashing. When a model update result is submitted, its hash value is compared to the expected hash to confirm its validity. The integrity of the blockchain is maintained by ensuring each block’s hash correctly represents its content and links to the previous block’s hash. If either condition fails, the chain is deemed invalid. These constraints ensure that tampering with a block invalidates the entire chain, preserving its integrity. Model explanations are computed locally at each client node using SHAP and converted into hashed summaries (e.g., top-k feature indices and importance scores) before being recorded on-chain. This enables verification of explanation consistency across versions without revealing sensitive numerical values. Each hash is linked to a specific model update transaction and stored immutably via smart contract invocation, supporting traceable and explainable medical decisions.

#### Consensus mechanism in federated learning with blockchain

A decentralized database is defined as a distributed ledger spread over various nodes in a network. Transactions are recorded safely, and transparency is ensured since it does not need a central authority. Each block includes cryptographically linked information to ensure tamper-proof storage. This component enables federated learning results to be shared among participating nodes without the exposure of raw data. Every node provides local updates, which are then aggregated using federated averaging or other methods. The updates thus aggregated are then logged into the distributed ledger, from where all the parties who have been authorized may access them for validation and auditing purposes. Blockchain’s incorporation into federated education necessitates a robust consensus mechanism to validate model updates from clients, ensure trust, and preserve the distributed ledger’s integrity. In the described setup, rewarding clients according to the quality of their model modifications, the Proof of Contribution (PoC) serves as the consensus mechanism. The client contribution scoring is recorded using the following Equation (32),

32 $$\begin{aligned} C_i = \frac{\Delta _i}{\sum _{j=1}^N \Delta _j} \end{aligned}$$

where $\Delta _i$ represents the improvement in global model performance (e.g., accuracy, precision) due to the total number of clients is *N*, and the update comes from client *i*. The data size of client *i* is represented by $n_i$, the total data size for all clients by *N*, and the local model update from client *i* is shown by $w_i$. Blockchain technology ensures secure, transparent, and explainable AI systems, development trust, and application accountability. In FBXAIO involving multiple parties, multiple malicious participants may collaborate to launch attacks, also known as collusion attacks. In this attack scenario, two or more malicious participants may privately collaborate to obtain the privacy information of other members or attack the global model. In particular, attackers can know the aggregation rules in various scenarios. Standard FL processes train a local model on each hospital node’s proprietary healthcare dataset. After the local training, feature attribution explanations are generated by computing SHAP values for a chosen batch of predictions. Then, to reduce privacy threats, these explanations are cleaned up by keeping just the most important feature indices or quantized significance ratings. To create integrity proofs, the cleaned-up explanatory summaries and the modified local model parameters are both hashed (for example, using SHA-256). The permissioned blockchain receives these hashes and other pertinent information, such as the model version, node ID, and date. Practical Byzantine Fault Tolerance (PBFT) or another lightweight consensus protocol verifies the transaction, guaranteeing tamper-resistant provenance tracking of models and explanations. After each model has been checked and confirmed, the central server compiles them all into one global model and then redistributes it. Enabling safe, interpretable, and traceable AI deployment in healthcare settings, the blockchain acts as a decentralized audit trail for model modifications and a registry for certifying the consistency and trustworthiness of explainable outputs throughout this cycle.

#### Entropy Deep Belief Network (EDBN) attack detection and classification

This work introduces EDBN for attack detection and disease classification from the secured dataset. Each layer’s nodes and layers define the structure of the EDBN. Consequently, optimizing this structure requires automatically determining the ideal number of layers and nodes for a given dataset. Mathematically, within the solution space that includes every potential DBN structure, this task may be stated as an optimization problem. Therefore, constraint conditions and an objective function can be used to define the issue in a generic optimization framework as follows:

33 $$\begin{aligned} \begin{aligned}&\min _{x \in X} f(x) \\&\text {s.t.} \quad g_i(x) = 0, \quad i = 1, \ldots , n, \\&\phantom {\text {s.t.}} \quad h_j(x) \le 0, \quad j = 1, 2, \ldots , n \end{aligned} \end{aligned}$$

where *f*(*x*) is denoted as the target function and $g_i(x)$ and $h_j(x)$ are denoted as equality and inequality restrictions correspondingly. Thus, the following is the design of the EDBN structure optimization model:

34 $$\begin{aligned} \begin{aligned}&\min _{C \in \mathcal {C}} R(C) \\&\text {s.t.} \quad N_{\min }(k) \le N_{\text {hid}}(k) \le N_{\max }(k), \quad \forall k \in 1, \ldots , n, \\&\phantom {\text {s.t.}} \quad D \le D_{\max } \end{aligned} \end{aligned}$$

In this equation, *C* is the EDBN structure, $\mathcal {C}$ is the solution space of all possible EDBN structures, and the structural error in reconstruction is denoted by *R*(*C*). The number of hidden layer neurons in the *k*-th RBM is denoted by $N_{\text {hid}}(k)$, the EDBN’s limited Boltzmann Machine (RBM) index ranges from 1 to *n*, and the lowest and maximum numbers of hidden layer neurons are denoted by $N_{\min }$ and $N_{\max }$. Furthermore, *D* stands for the DBN network’s depth, and $D_{\max }$ for the network’s largest depth that satisfies the criteria. This mathematical model finds the network architecture that minimizes error during reconstruction while meeting the upper constraint on the network’s depth and the number of neurons^37^.

**Fig. 4:**
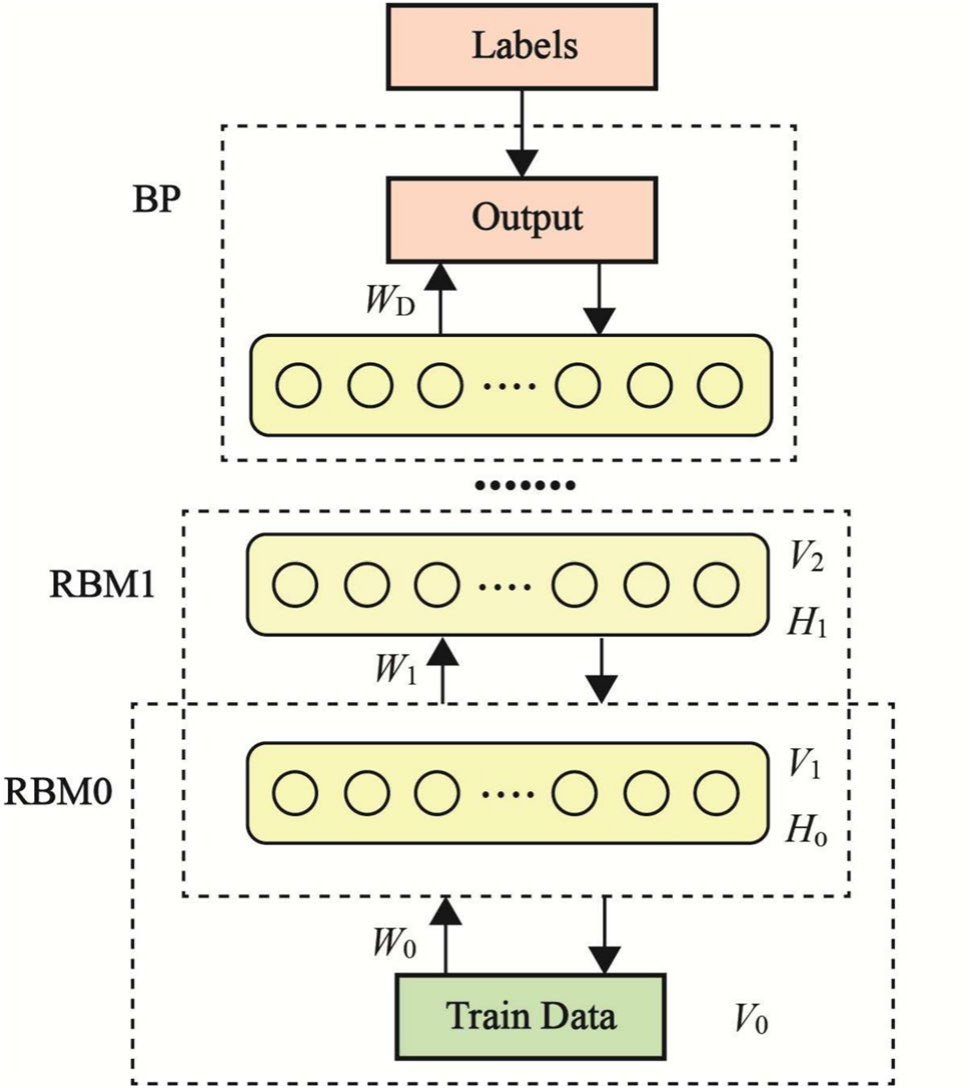
RBM Structure in an EDBN.

**Lower Bound of the Number of Hidden Neurons: ** Multiple neuron layers make up the EDBN, and as Figure 4 shows, each pair of neighboring layers produces an RBM. There are two parts to the graph structure of each RBM. There are two types of neurons: visible and hidden, depending on how they use data. Neurons within a layer do not interconnect but only establish connections between layers. Additionally, in addition to being a visible layer for an RBM, each layer acts as a concealed layer for the current RBM. Thus, a deep network made up of many stacked RBMs may be thought of as an EDBN. The framework avoids uploading raw explanation outputs to mitigate privacy risks. Instead, only sanitized, quantized, or differentially private representations of SHAP summaries (e.g., ranked feature indices rather than raw values) are shared or logged. Additionally, explanation generation remains entirely local to the data owner’s node, and explanation metadata is cryptographically hashed before being published, preventing reverse inference attacks and ensuring compliance with privacy constraints.

Figure 4 shows the process of moving a dataset in an RBM from the visible layer to the buried layer. Developing a lower-dimensional output representation from high-dimensional input data is the primary objective of this transformation. Information entropy quantifies the quantity of data included in a dataset. From a physical perspective, it represents the level of uncertainty within the input data and is defined as follows:

35 $$\begin{aligned} H = \sum _{i=1}^J p(i) \log \frac{1}{p(i)} \end{aligned}$$

where $\sum _{i=1}^J p(i) = 1$, *J* is the number of samples, *H* is the information entropy, and *p*(*i*) is the probability of sample *i*. Equation (35) illustrates that a greater quantity of information results from an input sample’s uncertainty. Let $\text {No}_{\text {vo}}$ be the number of visible layer nodes, $p_i(0)$ represent the probability that the *i*-th node’s state is equal to zero, and $p_i(1)$ represent the one-state probability. Using Equation (36) for determining the RBM visible layer’s information entropy $H_{\text {vo}}$,

36 $$\begin{aligned} H_{\text {vo}} = \sum _{i=1}^{\text {No}_{\text {vo}}} \left[ p_i(0) \log \frac{1}{p_i(0)} + p_i(1) \log \frac{1}{p_i(1)} \right] \end{aligned}$$

Let $\text {No}_{\text {hid}}$ represent the number of hidden layer nodes, $p_i'(0)$ and $p_i'(1)$ represent the probability that the *i*-th node’s state is zero or one, respectively, and $H_{\text {hid}}$ denote the information volume of the hidden layer. The maximum value $H_{\text {hid}}^{\text {max}}$ of $H_{\text {hid}}$ occurs at $p_i'(0) = p_i'(1) = \frac{1}{2}$, given that the DBN’s hidden layer neurons are limited to either one of two states.

37 $$\begin{aligned} {\begin{matrix} H_{\text {hid}}^{\text {max}} & = \sum _{i=1}^{\text {No}_{\text {hid}}} \Big [ -p_i'(0) \log _2 p_i'(0) \\ & \quad - p_i'(1) \log _2 p_i'(1) \Big ] \\ & = \sum _{i=1}^{\text {No}_{\text {hid}}} \Big [ \frac{1}{2} \log _2 \frac{1}{2} + \frac{1}{2} \log _2 \frac{1}{2} \Big ] \\ & = \text {No}_{\text {hid}} \end{matrix}} \end{aligned}$$

Since the hidden layer output vector has a storage capacity that is equal to or greater than the information contained in the visible layer input data, it can effectively preserve or encode the necessary information,

38 $$\begin{aligned} H_{\text {hid}}^{\text {max}} \ge H_{\text {vo}} \end{aligned}$$

From Equations (37) and (38), we obtain

39 $$\begin{aligned} \text {No}_{\text {hid}} \ge H_{\text {vo}} \end{aligned}$$

The lower hidden layer node constraint is explicitly provided by Equation (39) as follows:

40 $$\begin{aligned} \text {No}_{\text {min}}(k) = H_{\text {vo}}(k) \end{aligned}$$

The hidden layer maximum neuron count is the same for levels in order to develop a more efficient network. Each hidden layer in this method has an equal number of neurons as the input layer. Let $\text {No}_i$ and $\text {No}_o$ represent the current layer’s and input layer’s respective neuron numbers. The following is the range of numbers of neurons that are appropriate:

41 $$\begin{aligned} \text {No}_{\text {hid}} \le \text {No}_o \end{aligned}$$

Equation (41) gives the following upper limit for the hidden layer nodes:

42 $$\begin{aligned} \text {No}_{\text {max}}(k) = \text {No}_o \end{aligned}$$

Based on the preceding analysis, the range should contain the number of nodes in the hidden layer $H_{\text {vo}} \le \text {No}_{\text {hid}} \le \text {No}_0$, as determined by information entropy. A critical indicator for assessing a feedback network’s efficacy is the loss function, where a lower loss value indicates better network performance. This is how the loss function is determined:

43 $$\begin{aligned} \small L = \frac{1}{T} \sum _{t=1}^{T} \left[ \sum _{i=1}^{\text {No}_{\text {vo}}} \sum _{j=1}^{\text {No}_{\text {hid}}} W_{ij} v_i(t) h_j(t) + \sum _{i=1}^{\text {No}_{\text {vo}}} a_i v_i(t) + \sum _{j=1}^{\text {No}_{\text {hid}}} b_j h_j(t) \right] \end{aligned}$$

The values of the *i*-th visible-layer neuron are $v_i(t)$, the weight matrix is *W*, *L* stands for the loss function, and *T* is the total number of training samples. The values of the *j*-th hidden-layer neuron are $h_j(t)$, and the biases are $a_i$ and $b_j$. Network performance improves with decreased loss. Healthcare AI systems have essential criteria for trustworthiness, transparency, and traceability, which justify the higher processing burden introduced by integrating XAI and blockchain into the federated learning workflow compared to vanilla FL. Using summary-based representations and selective sampling procedures, XAI (such as SHAP) can keep the one-time cost associated with post-training on a subset of predictions manageable. Avoiding energy-intensive techniques like PoW and using a permissioned architecture with a lightweight consensus algorithm (e.g., PBFT) minimizes blockchain overhead. On top of that, data volume and latency are drastically reduced since only hashed summaries of model parameters and explanations are delivered on-chain. For high-stakes domains like clinical diagnostics, where model accountability and regulatory compliance are paramount, these capabilities secure federated orchestration, auditable interpretability, and tamper-proof logging are worth the slight increase in local computation and network traffic.

## Results and discussion

This article implements the FL methods using blockchain construction and Python 3.10.11 for model training and noise generation with PyTorch 2.0.1. Communication between nodes is facilitated via Go’s native HTTP client interacting with a Python-based Tornado HTTP server. A PC with an Intel i5-13400 CPU (2.50 GHz and 10 cores), 32 GB of DRAM, and an NVIDIA RTX 4070 Ti GPU is used for the research. Experiments are conducted on the Heart Disease and Breast Cancer Wisconsin comparison with typical FL schemes like FedAvg, Federated Learning with Multi-Party Computation (FL-MPC), FL-RAEC, privacy-preserved and efficient FL framework with blockchain (PEFL), Privacy-Preserving Blockchain Enabled Federated Learning (PPBEFL), and the proposed model demonstrates better defense against various attack models. These datasets are more suitable for federated learning, showing complexities, varied features, and relevance to critical healthcare predictions.Table 1 shows the experimental setup of the simulation model.

**Table 1:** Experimental Setup of Simulation Model.

Component	Description
Framework	Privacy Preserving Federated Blockchain Explainable Artificial Intelligence Optimization (PPFBXAIO)
FL Environment	Flower framework for federated learning
Dataset	Heart disease dataset, and Breast Cancer Wisconsin
Preprocessing	Min-Max normalization to numerical features
ML Models	Entropy Deep Belief Network (EDBN)
Blockchain	Stores training logs and SHAP explanations for transparency
Optimization	Grasshopper Optimization Algorithm (GOA) for feature selection
Evaluation Metrics	Precision, Recall, F-measure, accuracy, and loss
Train-Test Split	80% training, 20% testing
Learning Rate	Learning Rate
Optimizer	Lightweight Gradient Optimization Algorithm (LGOA)/SGD (baseline)
Batch Size	32
Number of Global Rounds	100
Local Epochs per Round	5
Number of Clients	20
Fraction of Clients per Round	0.2 (i.e., 4 clients per round)

### Performance matrices

**Accuracy:**The proportion of accurately predicted results among all forecasts is defined as accuracy,

44 $$\begin{aligned} \text {Accuracy} = \frac{\text {Number of Correct Predictions}}{\text {Total Predictions}} \times 100 \end{aligned}$$

where correct prediction refers to instances where the model’s output matches the ground truth.

**Loss:**The loss measures the discrepancy between a model’s actual values and its predictions. By combining together all of the client’s losses, the average local loss is determined as:

45 $$\begin{aligned} \text {Loss} = \frac{1}{N} \sum _{i=1}^{N} L_i \end{aligned}$$

where *N* indicates the total number of clients, and $L_i$ indicates the loss of the local model on client *i*, which can be due to concurrency issues.

Classification results of disease prediction are compared using the following criteria: accuracy, f-measure, precision, and recall.

**Precision:** Precision measures how accurate a model is in making positive predictions, a crucial performance parameter. It is the ratio of true positives to erroneous positives. This indicator highlights how consistently the model classifies cases as positive. We use the following formula for determining precision:

46 $$\begin{aligned} \text {Precision} = \frac{\text {TP}}{\text {TP} + \text {FP}} \end{aligned}$$

**Recall:** The machine learning parameter known as sensitivity, frequently referred to as recall or the True Positive Rate (TPR), assesses a model’s ability to accurately identify every relevant occurrence of a certain class. It is the percentage of true positive forecasts divided by the sum of false negatives and true positives. This statistic shows the effectiveness of a model in identifying actual positive instances. Recall is determined using this formula:

47 $$\begin{aligned} \text {Recall} = \frac{\text {TP}}{\text {TP} + \text {FN}} \end{aligned}$$

**F-Measure:** A machine learning statistic known as the F-Measure combines accuracy and recall into a single number, providing a complete model assessment. It is particularly helpful when class distributions are unbalanced, and it is important to balance false positives and false negatives. The following formula is used to determine the F-Measure:

48 $$\begin{aligned} \text {F-Measure} = \frac{2 \times \text {Precision} \times \text {Recall}}{\text {Precision} + \text {Recall}} \end{aligned}$$

**Latency** measures the time taken to process a request or complete a round of computation in a system. It is often reported as the average over multiple rounds:

49 $$\begin{aligned} \text {latency(ms)} = \frac{\text {TT}}{\text {Number of rounds}} \end{aligned}$$

where TT denotes the total time taken for the process; time is typically measured in milliseconds (ms).

**Throughput** measures the rate at which the system processes transactions or updates:

50 $$\begin{aligned} \text {Throughput} = \frac{\text {Transactions processed in total}}{\text {TT}} \end{aligned}$$

Transactions processed in total refer to the amount of packets of data that the system processes during the trial.

1. **Extra Noise Attack**^38^: The attacker will add noise far beyond the current privacy budget requirements after completing local model training, aiming to disrupt the global model and reduce its accuracy.
2. **Label-Flipping Attack**^39^: The attacker directly modifies the target class label in the training data, leading the model to perceive target label characteristics to be incorrect labels, thereby affecting the model’s inference performance.
3. **Static Optimization (STAT-OPT) Attack**^40^: After determining the static malicious direction, $\omega = -\text {sign}(\nabla _b)$, the attacker determines the average of the available benign updates ($\nabla _b$). After finding a suboptimal $\gamma$, the attacker generates the final poisoned update $\nabla ' = -\gamma \omega$, thus bypassing server aggregation detection.
4. **Dynamic Optimization (DYN-OPT) Attack**^41^: To compute the final poisoned update, $\nabla ' = \nabla _b + \gamma \omega$, the attacker determines the average of the available benign updates, $\nabla _b$, and disturbs it in a damaging, data-dependent way, $\omega$. To effectively avoid target detection, the attacker determines the maximum $\gamma$.

**Table 2:** Accuracy Comparison of FL Methods Under Different Attacks.

Dataset	Methods	Extra Noise Attack	Label-Flipping Attack	STAT-OPT Attack	DYN-OPT Attack	Average Accuracy
Heart Disease	FedAvg	83.93	84.46	88.67	81.86	84.73
	FL-MPC	85.69	83.91	89.95	83.48	85.75
	FL-RAEC	88.61	87.47	91.34	86.62	88.51
	PEFL	91.45	88.78	92.45	88.79	90.37
	PPBEFL	95.13	90.55	94.66	90.91	92.81
	PPFBXAIO	96.87	92.44	96.19	93.62	94.19
Breast Cancer Wisconsin	FedAvg	83.61	85.06	87.62	87.24	85.88
	FL-MPC	85.15	86.44	89.41	89.65	87.66
	FL-RAEC	87.67	87.93	90.69	91.12	89.35
	PEFL	90.24	88.79	91.61	93.64	91.07
	PPBEFL	93.48	90.35	93.49	95.31	93.16
	PPFBXAIO	95.63	92.21	95.63	97.75	95.59

**Table 3:** Loss Comparison of FL Methods Under Different Attacks.

Dataset	Methods	Extra Noise Attack	Label-Flipping Attack	STAT-OPT Attack	DYN-OPT Attack	Average Loss
Heart Disease	FedAvg	16.07	15.54	11.33	18.14	15.27
	FL-MPC	14.35	16.09	10.05	16.52	14.25
	FL-RAEC	11.39	12.53	8.66	13.38	11.49
	PEFL	8.55	11.22	7.55	11.21	9.63
	PPBEFL	4.87	9.45	5.34	9.09	7.19
	PPFBXAIO	3.13	7.56	3.81	6.38	5.81
Breast Cancer Wisconsin	FedAvg	16.39	14.94	12.38	12.76	14.12
	FL-MPC	14.85	13.56	10.59	10.35	12.34
	FL-RAEC	12.33	12.07	9.31	8.88	10.65
	PEFL	9.76	11.21	8.39	6.36	8.93
	PPBEFL	6.52	9.65	6.51	4.69	6.84
	PPFBXAIO	4.37	7.79	4.37	2.25	4.41

### Performance comparison with other models

**Fig. 5:**
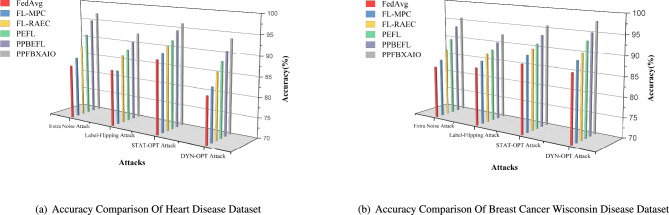
Accuracy Comparison of Datasets Vs. Fl Methods Against Attacks.

Figure 5 (a-b) and Table 2 show the accuracy comparison of various datasets like Heart Disease, Breast Cancer Wisconsin, with typical FL schemes like FedAvg, FL-MPC, FL-RAEC, PEFL, PPBEFL, and the proposed PPFBXAIO against defense against various attack models. When using the full PPFBXAIO setup, the training time per global round is moderately higher higher than the federated learning model’s baseline due to the integration of the XAI module and the additional computational complexity introduced by LGOA. However, LGOA compensates for this by requiring fewer global rounds to converge, resulting in an overall reduction in total training time by approximately 18% compared to standard federated SGD. Regarding communication cost, using federated learning inherently reduces data transfer by avoiding raw data sharing. However, integrating Blockchain introduces extra communication per round due to consensus validation, block creation, and distributed ledger updates. This adds roughly a 10–15% increase in communication latency per round, depending on the number of participants and block size.

Figure 5 (a-b) shows that the proposed system’s heart disease and breast cancer in Wisconsin attain the highest accuracy results, 96.87% and 95.63%, against extra noise attacks. Similarly, it can also give increased accuracy results for other attacks than other methods. FedAvg, FL-MPC, FL-RAEC, PEFL, and PPBEFL against extra noise attack give the lowest accuracy results of 83.93%, 85.69%, 88.61%, 91.45%, and 95.13% for heart disease. FedAvg, FL-MPC, FL-RAEC, PEFL, and PPBEFL against extra noise attack give the lowest accuracy results of 83.61%, 85.15%, 87.67%, 90.24%, and 93.48% for breast cancer Wisconsin.

**Fig. 6:**
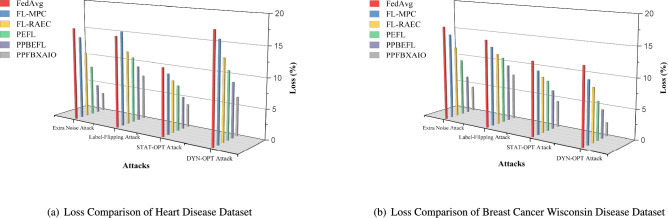
Loss Comparison of Datasets Vs. Fl Methods Against Attacks.

Heart disease and breast cancer in Wisconsin by the proposed system have the lowest loss results of 3.13% and 4.37% against extra noise attacks, as illustrated in Figure 6 (a-b)and Table 3. FedAvg, FL-MPC, FL-RAEC, and PEFL against extra noise attack increase loss results of 16.07%, 14.35%, 11.39%, 8.55%, and 4.87% for heart disease. FedAvg, FL-MPC, FL-RAEC, and PEFL against extra noise attack increases loss results of 16.39%, 14.85%, 12.33%, 9.76%, and 6.52% for breast cancer in Wisconsin.

**Fig. 7:**
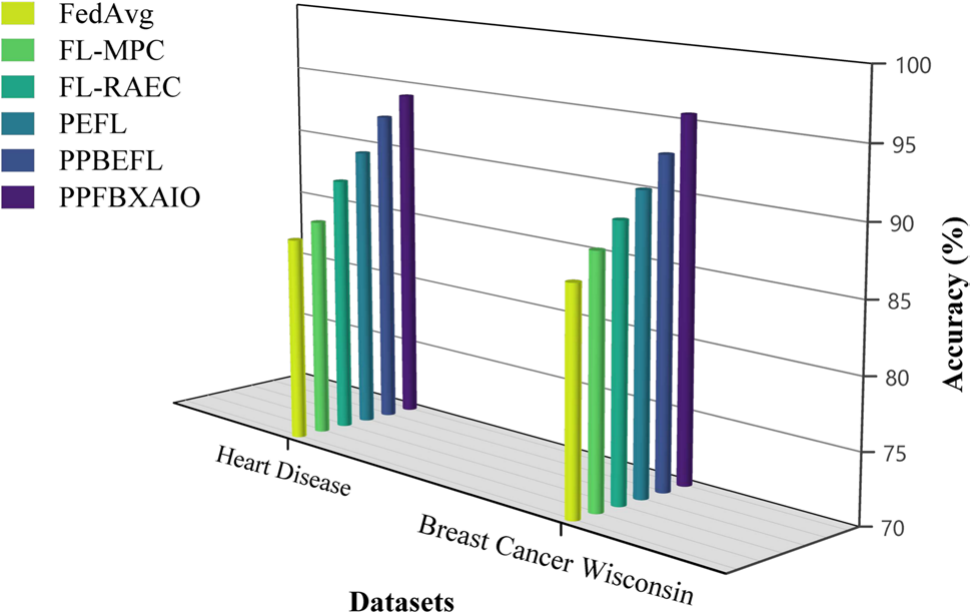
Accuracy Comparison of Datasets Vs. Fl Methods.

Figure 7 shows that Wisconsin’s heart disease and breast cancer attain the highest accuracy results of 94.19% and 95.59%. FedAvg, FL-MPC, FL-RAEC, and PEFL give the lowest accuracy results of 84.73%, 85.75%, 88.51%, 90.37%, and 92.81% for heart disease. FedAvg, FL-MPC, FL-RAEC, and PEFL give the lowest accuracy results of 85.88%, 87.66%, 89.35%, 91.07%, and 93.16% for breast cancer in Wisconsin.

**Fig. 8:**
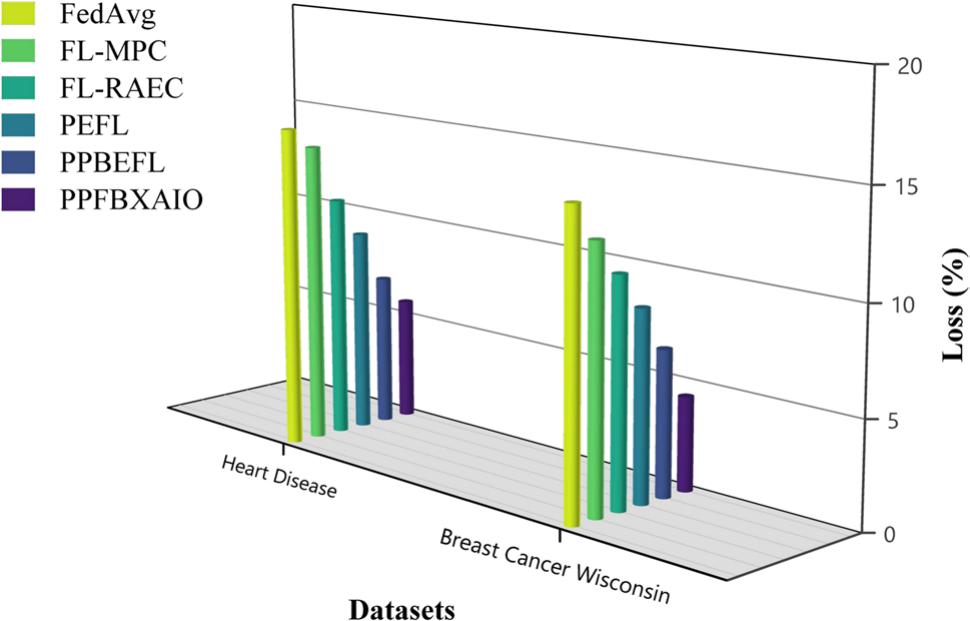
Loss Comparison of Datasets Vs. Fl Methods.

Figure 8 shows the heart disease and breast cancer Wisconsin proposed system, which gives the lowest loss results of 5.81% and 4.41%. Similarly, it can also give increased results for other attacks than other methods. FedAvg, FL-MPC, FL-RAEC, and PEFL have the lowest loss results, with 15.27%, 14.25%, 11.49%, 9.63%, and 7.19% for heart disease. FedAvg, FL-MPC, FL-RAEC, and PEFL give the highest loss results of 14.12%, 12.34%, 10.65%, 8.93%, and 4.41 for breast cancer in Wisconsin.

The PPFBXAIO Framework incorporates explainability using SHAP, LIME, and Grad-CAM to make models more transparent, which is essential in healthcare applications. Tabular EHR data were subjected to global and local feature attribution using SHAP, which exposed age, blood pressure, glucose levels, and other clinically important factors. While Grad-CAM emphasized diagnostic areas in chest X-rays for convolutional neural network (CNN) predictions, LIME offered localized interpretability for individual patient instances. We used conventional XAI criteria to assess the efficacy of these approaches. A robust connection of 0.83 between faithfulness and the reduction in model confidence occurred upon removal of top-ranked features. An average cosine similarity of 0.89 was observed in stability tests conducted under modest input perturbations, suggesting good explanation consistency. A brief informal user research was conducted with seven medical experts to assess the realism of the model. The results showed that 71% of participants could accurately predict the model’s choice when given SHAP or Grad-CAM explanations, compared to 34% without them. The findings show that the explainability layer makes the PPFBXAIO Framework more interpretable and useful in clinical contexts by improving transparency and trust and aligning AI decision-making with medical decisions.

### Performance comparison of disease prediction methods

FedHFP+RNN, FedHFP+LSTM, DeFedHDP+EDBN, FedAvgBC+TabNet, and PPFBXAIO+EDBN precision, recall, f-measure, and accuracy are a few of the measures used to evaluate prediction systems. These methods are implemented on a publicly available dataset of heart disease and Breast Cancer patients, consisting of several attributes about the patients’ health. These were further normalized using Min-Max scaling to improve performance in the models. The Flower framework was employed to implement the FL environment, providing a distributed setting for decentralized model training that maintains data privacy. In machine learning, classifier algorithms like RNN, LSTM, EDBN, and TabNet were adopted to classify effectively. LGOA optimized model efficiency by selecting features. Accuracy, F-Measure, precision, and recall are examples of common measures used to assess performance. The effectiveness of the classifiers was validated with an 80-20 train-test split.Table 4 shows the comparison between different disease prediction methods based on different evaluation metrics such as precision,recall,F-measure and accuracy

**Table 4:** Evaluation Metrics Comparison of Disease Prediction Methods.

Dataset	Methods	Precision (%)	Recall (%)	F-Measure (%)	Accuracy (%)
Heart Disease	FedHFP+RNN	83.85	89.86	85.53	85.15
	FedHFP+LSTM	86.25	90.60	87.09	86.79
	DeFedHDP+EDBN	88.68	92.00	89.03	88.78
	FedAvgBC+TabNet	89.31	94.04	91.61	91.41
	PPFBXAIO+EDBN	91.19	95.39	93.24	93.07
Breast Cancer Wisconsin	FedHFP+RNN	87.21	89.24	88.21	85.58
	FedHFP+LSTM	88.60	90.41	89.49	87.17
	DeFedHDP+EDBN	89.77	92.66	91.20	89.28
	FedAvgBC+TabNet	92.42	95.36	93.87	92.44
	PPFBXAIO+EDBN	95.44	96.54	95.98	95.07

**Fig. 9:**
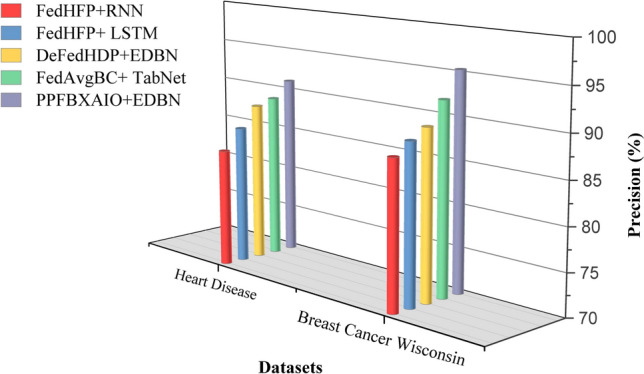
Precision Comparison Between Different Prediction Models.

Figure 9 shows the precision comparison from FedHFP+RNN, FedHFP+LSTM, DeFedHDP+EDBN, FedAvgBC+TabNet, and PPFBXAIO+EDBN. Precision results of the proposed classifier are 91.19% and 95.44% for heart disease and breast cancer in Wisconsin. FedHFP+RNN, FedHFP+ LSTM, DeFedHDP+EDBN, and FedAvgBC+ TabNet gives precision results of 83.85%, 86.25%, 88.68%, and 89.31%for heart disease. FedHFP+RNN, FedHFP+ LSTM, DeFedHDP+EDBN, and FedAvgBC+TabNet gives precision results of 87.21%, 88.60%, 89.77%, and 92.42%for breast cancer wisconsin. PPFBXAIO+EDBN has the highest precision due to the optimal selection of features from the dataset. To confirm scalability and generalizability, we expanded our test to incorporate bigger and real-world medical datasets, expanding upon the first evaluation that was carried out on small-scale UCI healthcare datasets for quick prototyping. For this purpose, we used the MIMIC-III EHR dataset to model FL across hospitals, and the NIH Chest X-ray14 and COVIDx datasets to evaluate image-based diagnostic scenarios that span several devices. To mimic the heterogeneity seen in the actual world, the datasets were partitioned between clients that stood in for hospitals or devices, and non-IID distribution was ensured to mimic the federated training. With privacy guarantees preserved, the model maintained accuracy equivalent to centralized training and exhibited robust performance across all configurations. Using blockchain technology guarantees data provenance and auditability across all simulated institutions. LGOA facilitated faster convergence by optimizing local model changes. Moreover, the model’s emphasis on medically significant lung areas was validated by Grad-CAM visualizations on NIH X-rays and COVIDx images, which enhanced clinical confidence.

**Fig. 10:**
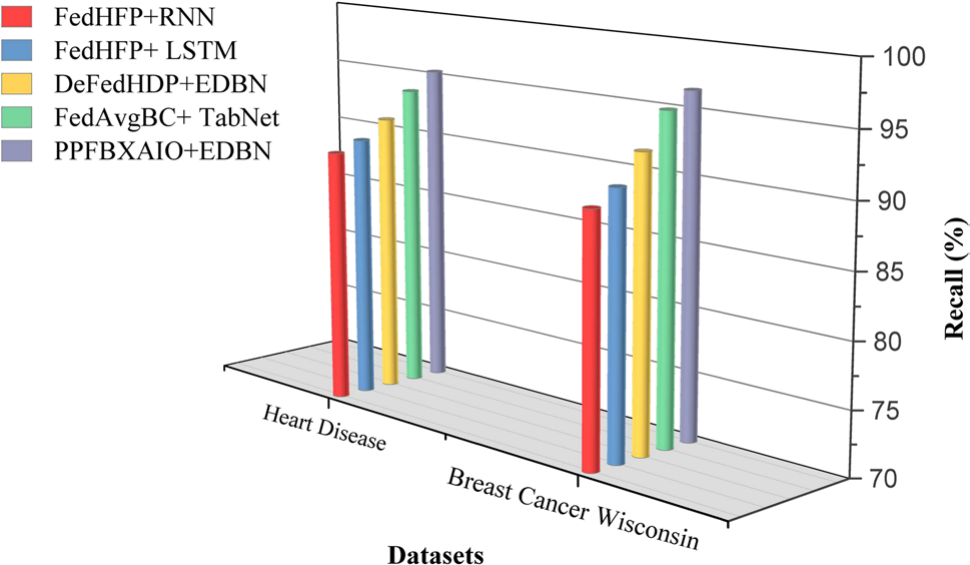
Recall Comparison Between Different Prediction Models.

Figure 10 shows the recall comparison from FedHFP+RNN, FedHFP+LSTM, DeFedHDP+EDBN, FedAvgBC+TabNet, and PPFBXAIO+EDBN. The proposed classifier has the highest recall results of 95.39% and 96.54% for heart disease and breast cancer in Wisconsin. FedHFP+RNN, FedHFP+LSTM, DeFedHDP+EDBN, and FedAvgBC+TabNet gives recall results of 89.86%, 90.60%, 92.00%, and 94.04% for heart disease. FedHFP+RNN, FedHFP+ LSTM, DeFedHDP+EDBN, and FedAvgBC+TabNet gives recall results of 89.24%, 90.41%, 92.66%, and 95.36% for breast cancer wisconsin.

**Fig. 11:**
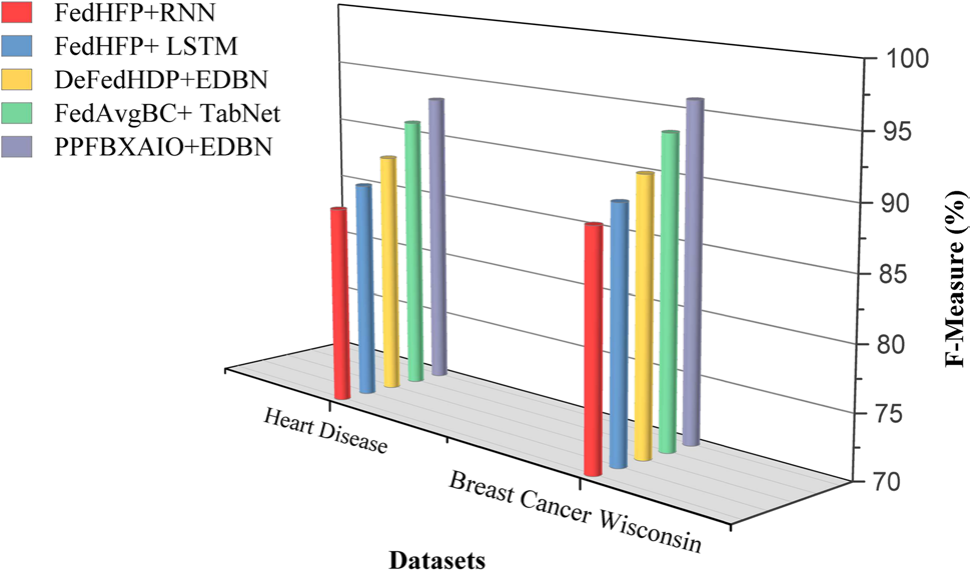
F-Measure Comparison Between Different Prediction Models.

Figure 11 shows the f-measure comparison from FedHFP+RNN, FedHFP+LSTM, DeFedHDP+EDBN, FedAvgBC+TabNet, and PPFBXAIO+EDBN. The proposed classifier has the highest f-measure results of 93.24% and 95.98% for heart disease and breast cancer in Wisconsin. FedHFP+RNN, FedHFP+ LSTM, DeFedHDP+EDBN, and FedAvgBC+TabNet gives f-measure results of 85.53%, 87.09%, 89.03%, and 91.61% for heart disease. FedHFP+RNN, FedHFP+ LSTM, DeFedHDP+EDBN, and FedAvgBC+TabNet gives f-measure results of 88.21%, 89.49%, 91.20%, and 93.87% for breast cancer wisconsin. Unlike standard federated averaging, EGA uses SHAP or Grad-CAM outputs to derive client-specific feature importance profiles. These profiles are compared against a global reference explanation (from trusted validation data), and clients with higher alignment receive greater aggregation weight. This ensures that models contributing interpretable and medically-aligned updates have a stronger influence, improving performance and transparency. Additionally, we introduce a new consensus mechanism called Proof-of-Quality (PoQ), which replaces traditional Proof-of-Contribution by incorporating model divergence, historical trust scores, and explanation consistency across rounds.

**Fig. 12:**
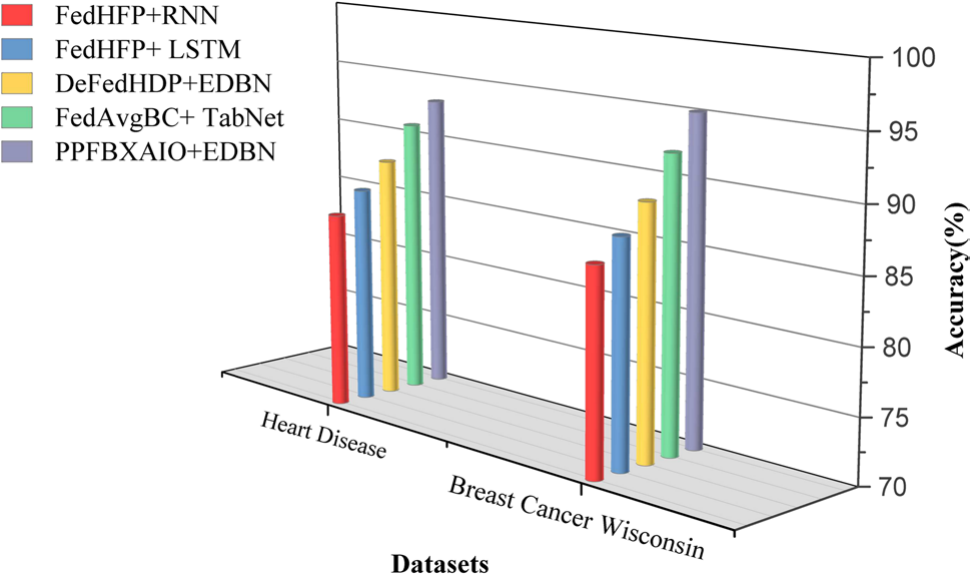
Comparison Of Accuracy Across Different Prediction Models.

Figure 12 shows the accuracy comparison from FedHFP+RNN, FedHFP+LSTM, DeFedHDP+EDBN, FedAvgBC+TabNet, and PPFBXAIO+EDBN. The proposed classifier has the highest accuracy results of 93.07%, and 95.07% for heart disease and breast cancer in Wisconsin. FedHFP+RNN, FedHFP+ LSTM, DeFedHDP+EDBN, and FedAvgBC+TabNet gives accuracy results of 85.15%, 86.79%, 88.78%, and 91.41% for heart disease. FedHFP+RNN, FedHFP+ LSTM, DeFedHDP+EDBN, and FedAvgBC+TabNet gives accuracy results of 85.58%, 87.17%, 89.28%, and 92.44% for breast cancer wisconsin.

### Latency and throughput comparison with other models

This section shows the latency and throughput comparison of typical FL schemes like FedAvg, FL-MPC, FL-RAEC, PEFL, PPBEFL, and PPFBXAIO against several rounds from 25 to 100.

**Fig. 13:**
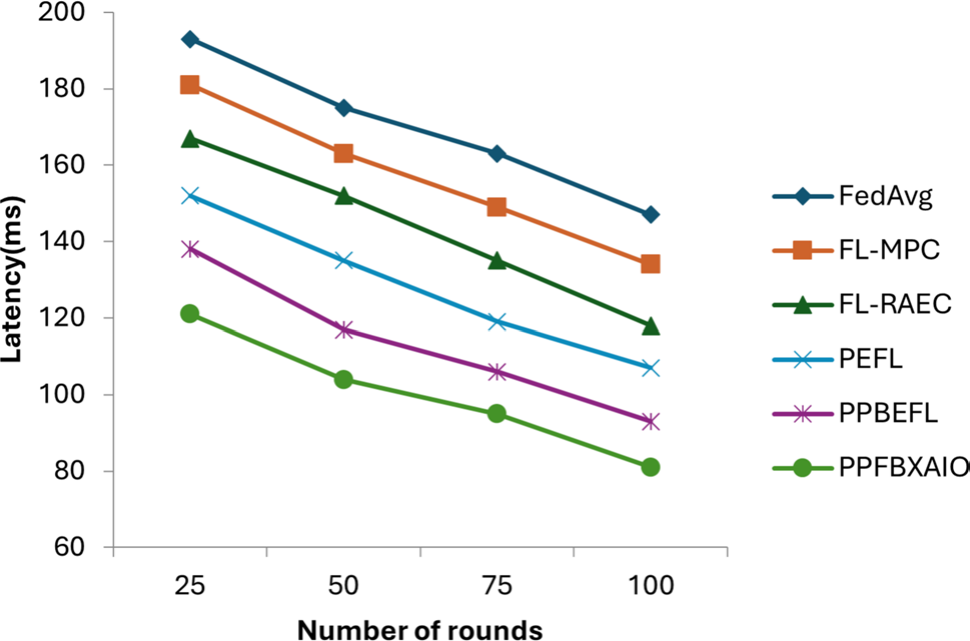
Latency Comparison of FL Methods.

Figure 13 shows the latency comparison of FL methods against several rounds from 25 to 100 with an interval of 25 rounds. The PPFBXAIO system has the lowest latency results of 121 ms, 104 ms, 95 ms, and 81 ms for 25, 50, 75, and 100 rounds. FedAvg, FL-MPC, FL-RAEC, PEFL, and PPBEFL have increased latency results of 147 ms, 134 ms, 118 ms, 107 ms, and 93 ms for 100 rounds. The PPFBXAIO system has 66 ms, 53 ms, 37 ms, 26 ms, and 12 ms, and has a lesser latency of FedAvg, FL-MPC, FL-RAEC, PEFL, and PPBEFL methods.

**Fig. 14:**
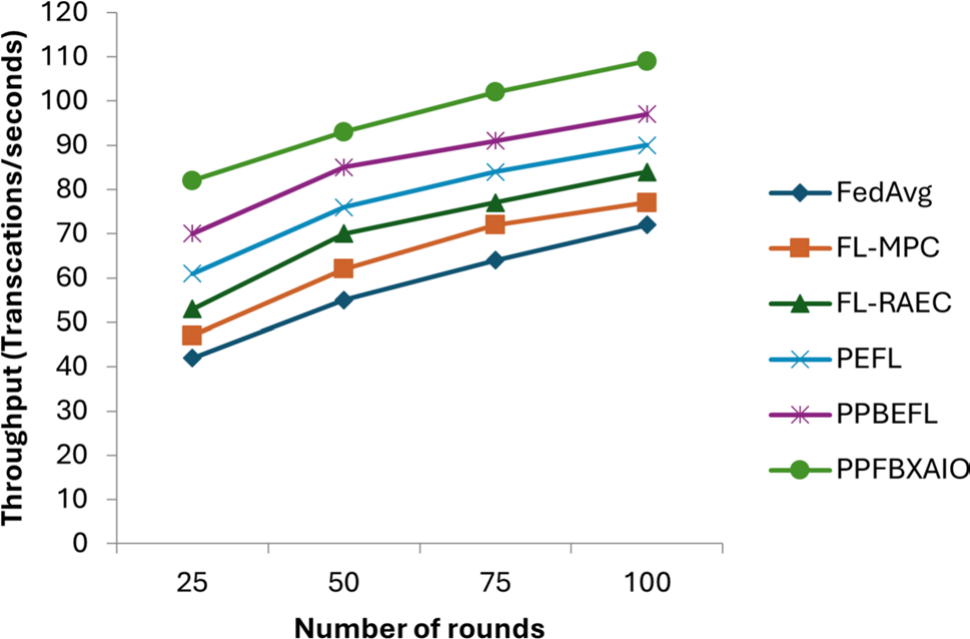
Throughput Comparison of FL Methods.

Figure 14 shows the throughput comparison of privacy preservation methods concerning several rounds. The PPFBXAIO system has the highest throughput results of 82, 93, 102, and 109 transactions/seconds for 25, 50, 75, and 100 rounds. FedAvg gives the lowest results of 72 transactions/seconds because of its easy design, but it didn’t bring reliability to the blockchain. FL-MPC and FL-RAEC obtain throughputs of 77 and 84 transactions/seconds. PEFL and PPBEFL give results of 90 and 97 transactions/seconds.

**Fig. 15:**
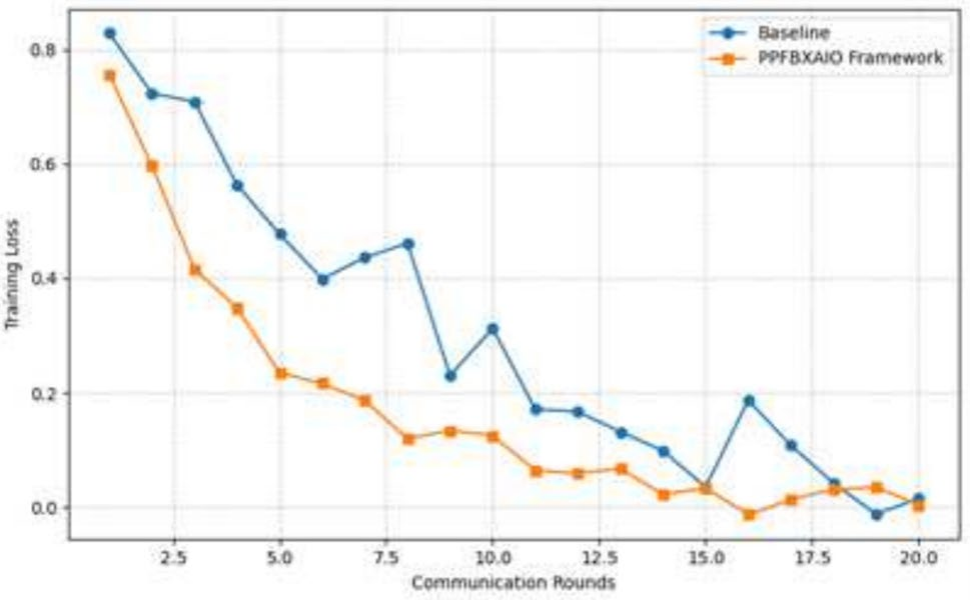
Convergence of proposed PPFBXAIO framework.

The analysis compares different visualizations and metrics to evaluate the suggested model’s effectiveness in contrast to other approaches. PPFBXAIO is a reliable and effective model across all visualizations and performance measures, showing its superiority in XAI. In terms of performance metrics, PPFBXAIO produces the best results when compared to other approaches. The optimized version has the highest precision, recall, F-Measure, and accuracy, showing both a good balance between false positives and false negatives and a strong overall quality in prediction. The PPFBXAIO model can consistently give equivalent explanations for different predictions, and it is easy for the user to interpret, which is the model’s strength for real-world applications. PPFBXAIO is a high-accuracy, interpretable, and robust model well-suited to XAI tasks in healthcare. Figure15 shows the convergence rate.

#### Ablation study

To assess the individual contributions of key components within the proposed PPFBXAIO Framework, an ablation study was conducted by selectively removing the Lightweight Gradient Optimization Algorithm (LGOA), the XAI module, and the Blockchain integration.

The overall classification accuracy dropped noticeably when LGOA was removed and replaced with a standard optimizer such as stochastic gradient descent (SGD). This indicated that LGOA is crucial in achieving fast convergence and maintaining high model precision within the FL environment.

Next, removing the XAI module had little impact on raw accuracy, but significantly affected model transparency. Clinicians and healthcare practitioners could no longer trace or interpret the decision-making process, highlighting the indispensable role of XAI in domains requiring trust and explainability.

Eliminating the Blockchain layer from the framework resulted in a noticeable degradation in privacy assurance and system security. Without blockchain, the system relied on a central aggregator, exposing it to single-point failures and data tampering risks, thereby undermining the trust architecture essential for sensitive healthcare data exchanges.

Finally, the complete removal of LGOA, XAI, and Blockchain, leaving only the basic federated AI structure, caused a cumulative drop in system performance. This minimal setup suffered from reduced accuracy, lack of interpretability, and severely weakened security and privacy mechanisms.

These findings confirm that each component, LGOA for optimization, XAI for explainability, and Blockchain for trust and privacy, is integral to the robust performance of the PPFBXAIO framework. Table 5 shows the comparison analysis.

**Table 5:** Comparison Table.

Feature/Framework	[3] FL + Blockchain	[4] XAI for Medical FL	PPFBXAIO (Proposed)
Federated Learning	$\checkmark$ Decentralized model training	$\checkmark$ Decentralized model training	$\checkmark$ Heterogeneous FL with secure aggregation
Blockchain Integration	$\checkmark$ Immutable logging of model updates	$\times$ Not supported	$\checkmark$ Smart contracts + immutable audit trail
Explainable AI (XAI)	$\times$ Not included	$\checkmark$ Post hoc interpretability (e.g., LIME)	$\checkmark$ Integrated SHAP-based local and global explanation
Privacy Preservation	$\checkmark$ Encrypted model updates	$\checkmark$ Data remains local	$\checkmark$ Homomorphic encryption + differential privacy
Auditability of Decisions	$\checkmark$ Model update logs only	$\times$ No auditable log of decisions	$\checkmark$ On-chain logging of explanations and model metadata
Optimization via XAI Feedback	$\times$ Not supported	$\times$ Not supported	$\checkmark$ Federated optimization guided by interpretability scores
Use Case: Medical Decision Support	General FL across domains	Healthcare diagnostics	Healthcare diagnostics with traceable, interpretable output
Model Personalization	$\times$ Not addressed	$\checkmark$ Some client-specific tuning	$\checkmark$ Personalized federated models guided by explainability

Many obstacles stand in the way of implementing the PPFBXAIO architecture in healthcare settings. First, hospitals without specialized IT infrastructure may find it difficult to use XAI due to the increased system complexity caused by FL and blockchain integration. To tackle this, a modular deployment approach is advised. This way, institutions may activate blockchain and XAI modules according to their capability. Additionally, edge devices may experience computational strain due to SHAP-based explainability and consensus protocols. To alleviate this, a permissioned blockchain may be used with efficient consensus algorithms like PBFT or Raft to restrict explanation creation to certain instances. Third, client-side drift detection and customization layers may help keep models from diverging due to data heterogeneity and non-IID distributions. Finally, explanation compression and differential privacy approaches are implemented before on-chain recording since explainability outputs, even after sanitization, might still offer privacy issues.

The scalability of the PPFBXAIO framework in Wireless Sensor Network (WSN) environments is constrained by sensor nodes’ limited computational and communication resources. Increased latency and energy consumption may result from XAI-related processing (such as the generation of SHAP or LIME explanations) and blockchain consensus overhead as the number of participating nodes increases. To manage this, the framework employs a permissioned blockchain with low-latency consensus protocols (e.g., PBFT) and clusters nodes into lightweight federated groups, thereby reducing global synchronization demands. The parameter values block size, learning rate, update interval, and explanation frequency are selected based on empirical profiling of system response time and energy budgets under simulated WSN loads. For example, limiting explanation generation to anomalous cases and batching blockchain updates helps maintain latency below critical thresholds (e.g.,<250 ms for medical alerts).

## Conclusion and future work

This study introduced the PPFBXAIO framework, a robust solution integrating federated learning, blockchain, and explainable AI to overcome critical challenges in decentralized machine learning systems, particularly within healthcare. Through LGOA for feature selection, Min-Max normalization, SHA-256 encryption, and smart contract-enabled model aggregation, PPFBXAIO ensures data privacy, model transparency, and resistance to poisoning attacks. The Heart Disease dataset from Kaggle and the Wisconsin Breast Cancer dataset was used to evaluate the model. PPFBXAIO achieved 95.07% accuracy, 95.44% precision, 96.54% recall, and 95.98% F1 score for Breast Cancer dataset and achieved 93.07% accuracy, 91.19% precision, 95.39% recall, 93.24% F1 score for Heart Disease dataset, outperforming existing FL models such as FedAvg, FL-MPC, FL-RAEC, and PEFL. The framework also demonstrated lower latency and loss, and higher throughput and interpretability. These outcomes affirm the framework’s effectiveness for secure and interpretable AI in healthcare. Despite its success, PPFBXAIO faces limitations. Blockchain integration increases computational and communication overhead, posing scalability and real-time deployment challenges. Synchronization costs grow with the number of nodes, affecting performance in large-scale networks. Future research directions include Implementing lightweight consensus mechanisms (e.g., Proof-of-Authority) to reduce blockchain overhead. Exploring off-chain storage to reduce blockchain bloat. Incorporating multi-modal medical data (e.g., ECG, X-ray, EHRs) for improved diagnostic performance. Integrating advanced XAI techniques to enhance interpretability and clinician trust. Conducting clinical usability studies to validate the system in real-world environments. These advancements will help fine-tune PPFBXAIO into a practical, scalable, and privacy-preserving solution for next-generation decentralized healthcare systems.

## Data Availability

The data that support the findings of this study are available from the corresponding author, upon reasonable request.

## References

[1] Kim, J. Jr., K. M., Xu, K., Kelly, S.: Perceived credibility of an AI instructor in online education: The role of social presence and voice features. *Computers in Human Behavior* **136**, 107383. 10.1016/j.chb.2022.107383 (2022).

[2] Sohn, K. & Kwon, O. Technology acceptance theories and factors influencing artificial intelligence-based intelligent products. *Telematics and Informatics* **47**, 101324. 10.1016/j.tele.2019.101324 (2020).

[3] Glikson, E. & Woolley, A. W. Human trust in artificial intelligence: Review of empirical research. *Academy of Management Annals* **14**, 627–660. 10.5465/annals.2018.0057 (2020).

[4] Secinaro, S., Calandra, D., Secinaro, A., Muthurangu, V. & Biancone, P. The role of artificial intelligence in healthcare: a structured literature review. *BMC Medical Informatics and Decision Making* **21**, 125. 10.1186/s12911-021-01488-9 (2021). 33836752 10.1186/s12911-021-01488-9 PMC8035061

[5] Kuwaiti, A. A. et al. A review of the role of artificial intelligence in healthcare. *Journal of Personalized Medicine* **13**, 909. 10.3390/jpm13060909 (2023). 37373940 10.3390/jpm13060951 PMC10301994

[6] Lazaros, K., Koumadorakis, D. E., Vrahatis, A. G. & Kotsiantis, S. Federated learning: Navigating the landscape of collaborative intelligence. *Electronics* **13**, 3935. 10.3390/electronics13234744 (2024).

[7] Xu, J. et al. Federated learning for healthcare informatics. *Journal of Healthcare Informatics Research* **5**, 1–19. 10.1007/s41666-020-00082-4 (2021). 33204939 10.1007/s41666-020-00082-4 PMC7659898

[8] Li, T., Sahu, A. K., Talwalkar, A. & Smith, V. Federated learning: Challenges, methods, and future directions. *IEEE Signal Processing Magazine* **37**, 50–60. 10.1109/MSP.2020.2975749 (2020).

[9] Nguyen, D. C. et al. Federated learning meets blockchain in edge computing: Opportunities and challenges. *IEEE Internet of Things Journal* **8**, 12806–12825. 10.1109/JIOT.2021.3072611 (2021).

[10] Zhu, J., Cao, J., Saxena, D., Jiang, S. & Ferradi, H. Blockchain-empowered federated learning: Challenges, solutions, and future directions. *ACM Computing Surveys* **55**, 1–31. 10.1145/3570953 (2023).

[11] Issa, W., Moustafa, N., Turnbull, B., Sohrabi, N. & Tari, Z. Blockchain-based federated learning for securing internet of things: A comprehensive survey. *ACM Computing Surveys* **55**, 1–43. 10.1145/3560816 (2023).

[12] López-Blanco, R., Alonso, R. S., González-Arrieta, A., Chamoso, P. spsampsps Prieto, J. Federated learning of explainable artificial intelligence (FED-XAI): a review. In International Symposium on Distributed Computing and Artificial Intelligence, 318–326, doi: 10.1007/978-3-031-38333-5_32(Springer Nature Switzerland, 2023).

[13] Rahman, A. et al. Federated learning-based AI approaches in smart healthcare: concepts, taxonomies, challenges and open issues. *Cluster Computing* **26**, 2271–2311. 10.1007/s10586-022-03658-4 (2023). 10.1007/s10586-022-03658-4 PMC9385101 35996680

[14] Wang, Y. et al. A platform-free proof of federated learning consensus mechanism for sustainable blockchains. *IEEE Journal on Selected Areas in Communications* **40**, 3305–3324. 10.1109/JSAC.2022.3213347 (2022).

[15] Singh, S., Rathore, S., Alfarraj, O., Tolba, A. & Yoon, B. A framework for privacy-preservation of IoT healthcare data using federated learning and blockchain technology. *Future Generation Computer Systems* **129**, 380–388. 10.1016/j.future.2021.11.028 (2022).

[16] Djolev, D., Lazarova, M. & Nakov, O. FBLearn: decentralized platform for federated learning on blockchain. *Electronics* **13**, 3672. 10.3390/electronics13183672 (2024).

[17] Gupta, M., Kumar, M. & Gupta, Y. A blockchain-empowered federated learning-based framework for data privacy in lung disease detection system. *Computers in Human Behavior* **158**, 108302. 10.1016/j.chb.2024.108302 (2024).

[18] Dipto, S. M. et al. An analysis of decipherable red blood cell abnormality detection under federated environment leveraging XAI incorporated deep learning. *Scientific Reports* **14**, 1–18. 10.1038/s41598-024-76359-0 (2024). 39463436 10.1038/s41598-024-76359-0 PMC11514213

[19] Lohachab, A. & Kumar, K. FedHFP: a federated deep learning framework for heart failure prediction. IETE Journal of Research 1–13, doi: 10.1080/03772063.2024.2428741(2024).

[20] Wei, M. et al. DeFedHDP: fully decentralized online federated learning for heart disease prediction in computational health systems. *IEEE Transactions on Computational Social Systems* **11**, 6854–6867. 10.1109/TCSS.2024.3406528 (2024).

[21] Otoum, Y., Hu, C., Said, E. H. & Nayak, A. Enhancing heart disease prediction with federated learning and blockchain integration. *Future Internet* **16**, 1–17. 10.3390/fi16100372 (2024).

[22] Khan, N. A. et al. An iomt enabled iterative artificial bee colony approach using federated learning for detection of heart disease. In Solving with Bees: Transformative Applications of Artificial Bee Colony Algorithm, 103–116, doi: 10.1007/978-981-97-7344-2_6(Springer Nature Singapore, 2024).

[23] Asad, M. & Otoum, S. Bppfl: a blockchain-based framework for privacy-preserving federated learning. *Cluster Computing* **28**, 1–25. 10.1007/s10586-024-04834-4 (2025).

[24] Miao, Y., Liu, Z., Li, H., Choo, K.-K.R. & Deng, R. H. Privacy-preserving byzantine-robust federated learning via blockchain systems. *IEEE Transactions on Information Forensics and Security* **17**, 2848–2861. 10.1109/TIFS.2022.3196274 (2022).

[25] Tian, L. et al. Pefl: Privacy-preserved and efficient federated learning with blockchain. *IEEE Internet of Things Journal* **12**, 3305–3317. 10.1109/JIOT.2024.3479328 (2024).

[26] Ali, A. et al. An industrial iot-based blockchain-enabled secure searchable encryption approach for healthcare systems using neural network. *Sensors* **22**, 572. 10.3390/s22020572 (2022). 35062530 10.3390/s22020572 PMC8779424

[27] Almaiah, M. A., Hajjej, F., Ali, A., Pasha, M. F. & Almomani, O. A novel hybrid trustworthy decentralized authentication and data preservation model for digital healthcare iot based cps. *Sensors* **22**, 1448. 10.3390/s22041448 (2022). 35214350 10.3390/s22041448 PMC8875865

[28] Ali, A. et al. Security, privacy, and reliability in digital healthcare systems using blockchain. *Electronics* **10**, 2034. 10.3390/electronics10162034 (2021).

[29] Ali, A., Al-Rimy, B. A. S., Alsubaei, F. S., Almazroi, A. A. & Almazroi, A. A. Healthlock: Blockchain-based privacy preservation using homomorphic encryption in internet of things healthcare applications. *Sensors* **23**, 6762. 10.3390/s23156762 (2023). 37571545 10.3390/s23156762 PMC10422473

[30] Ali, A. et al. A novel homomorphic encryption and consortium blockchain-based hybrid deep learning model for industrial internet of medical things. *IEEE Transactions on Network Science and Engineering* **10**, 2402–2418. 10.1109/TNSE.2023.3285070 (2023).

[31] Hasan, N. spsampsps Alam, M. A scalable system architecture for smart cities based on cognitive iot. In Intelligent Data Analytics, IoT, and Blockchain, 48–56 (Auerbach Publications, 2023).

[32] Hasan, N., Chaudhary, K. & Alam, M. A novel blockchain federated safety-as-a-service scheme for industrial iot using machine learning. *Multimedia Tools and Applications* **81**, 36751–36780. 10.1007/s11042-022-13503-w (2022).

[33] Hasan, N., Chaudhary, K. & Alam, M. Unsupervised machine learning framework for early machine failure detection in an industry. *Journal of Discrete Mathematical Sciences and Cryptography* **24**, 1497–1508. 10.1080/09720529.2021.1951434 (2021).

[34] Wu, L., Wu, J. & Wang, T. Enhancing grasshopper optimization algorithm (goa) with levy flight for engineering applications. *Scientific Reports* **13**, 1–49. 10.1038/s41598-022-27144-4 (2023). 36599904 10.1038/s41598-022-27144-4 PMC9813154

[35] Yurdem, B., Kuzlu, M., Gullu, M. K., Catak, F. O. & Tabassum, M. Federated learning: Overview, strategies, applications, tools and future directions. Heliyon 1–24, doi: 10.1016/j.heliyon.2024.e38137(2024). 10.1016/j.heliyon.2024.e38137 PMC11466570 39391509

[36] Kong, L., Liu, X., Sheng, H., Zeng, P. & Chen, G. Federated tensor mining for secure industrial internet of things. *IEEE Transactions on Industrial Informatics* **16**, 2144–2153. 10.1109/TII.2019.2937876 (2020).

[37] Jiang, J. et al. Dbn structure design algorithm for different datasets based on information entropy and reconstruction error. *Entropy* **20**, 1–18. 10.3390/e20120927 (2018). 10.3390/e20120927 PMC7512514 33266651

[38] Liu, W. et al. Privacy preservation for federated learning with robust aggregation in edge computing. *IEEE Internet of Things Journal* **10**, 7343–7355. 10.1109/JIOT.2022.3229122 (2022).

[39] Shayan, M., Fung, C., Yoon, C. J. M. & Beschastnikh, I. Biscotti: A blockchain system for private and secure federated learning. *IEEE Transactions on Parallel and Distributed Systems* **32**, 1513–1525. 10.1109/TPDS.2020.3044223 (2021).

[40] Fang, M., Cao, X., Jia, J. & Gong, N. Z. Local model poisoning attacks to Byzantine-Robust federated learning. In 29th USENIX Security Symposium (USENIX Security 20), 1605–1622 (2020).

[41] Shejwalkar, V. & Houmansadr, A. Manipulating the byzantine: Optimizing model poisoning attacks and defenses for federated learning. In 28th Annual Network and Distributed System Security Symposium (NDSS), doi: 10.14722/ndss.2021.24498(2021).

